# The effectiveness of anti-inflammatory and anti-seizure medication for individuals with single enhancing lesion neurocysticercosis: A meta-analysis and expert group-based consensus recommendations

**DOI:** 10.1371/journal.pntd.0009193

**Published:** 2021-03-31

**Authors:** Annette Abraham, Javier A. Bustos, Hélène Carabin, Robert de Meijere, Priyadarshi S. Sahu, Vedantam Rajshekhar, Gagandeep Singh, A. Clinton White, Peter L. Chiodini, Sarah Gabriël, Mamoun Homeida, Theodore Nash, Bernard Ngowi, Xiao Nong Zhou, Christina Coyle, Hector H. Garcia, Andrea S. Winkler

**Affiliations:** 1 Department of Neurology, Center for Global Health, School of Medicine, Technical University of Munich, Munich, Germany; 2 Centre for Global Health, Department of Community Medicine and Global Health, Institute of Health and Society, University of Oslo, Oslo, Norway; 3 Center for Global Health, Universidad Peruana Cayetano Heredia, Lima, Peru, and Cysticercosis Unit, Instituto Nacional de Ciencias Neurológicas, Lima, Peru; 4 Département de Pathologie et de Microbiologie, Faculté de Médecine Vétérinaire, Université de Montréal, Saint-Hyacinthe, Canada; 5 Département de médecine sociale et préventive, École de santé publique, université de Montréal, Montréal, Canada; 6 Centre de Recherche en Santé Publique de l’Université de Montréal et du Centre Intégré Universitaire de Santé et des Services Sociaux de sud de l’île de Montréal, Montréal, Canada; 7 Groupe de recherche en épidémiologie des zoonoses et santé publique (GREZOSP), Montréal, Canada; 8 Department of Microbiology & Immunology, Medical University of the Americas, Nevis, West Indies; 9 Department of Neurological Sciences, Christian Medical College, Vellore, India; 10 Dayanand Medical College, Ludhiana, India; 11 Infectious Disease Division, Department of Internal Medicine, University of Texas Medical Branch, Galveston, TX, United States of America; 12 Hospital for Tropical Diseases and the London School of Hygiene and Tropical Medicine, London, United Kingdom; 13 Department of Veterinary Public Health and Food Safety, Faculty of Veterinary Medicine, Ghent University, Belgium; 14 University of Medical Sciences and Technology, Khartoum, Sudan; 15 Laboratory of Parasitic Diseases, National Institutes of Allergy and Infectious Diseases, National Institute of Health, United States of America; 16 National Institute for Medical Research, Muhimbili Medical Research Centre, Dar es Salaam, Tanzania; 17 University of Dar es Salaam, Mbeya College of Health and Allied Sciences, Mbeya, Tanzania; 18 National Institute of Parasitic Diseases, Chinese Center for Disease Control and Prevention, Shanghai, China; 19 Albert Einstein College of Medicine, Bronx, NY, United States of America; Federal University of Ceará, Fortaleza, Brazil, BRAZIL

## Abstract

Single brain enhancing lesions (SEL) are the most common presentation of neurocysticercosis (NCC) observed on neuroimaging in people presenting with epileptic seizures not only on the Indian sub-continent and in travelers returning from cysticercosis-endemic regions, but are also present in other parts of the world.

The aim of this study, which consisted of a systematic review (CRD42019087665), a meta-analysis and an expert group consultation, was to reach consensus on the best anti-seizure medication and anti-inflammatory treatment for individuals with SEL NCC.

Standard literature review methods were used. The Cochrane risk of bias tool was used and random effects model meta-analyses were performed. The quality of the body of evidence was rated using GRADE tables. The expert committee included 12 gender and geographically balanced members and recommendations were reached by applying the GRADE framework for guideline development.

The 1–1.5-year cumulative incidence of seizure recurrence, cyst resolution or calcification following anti-seizure medication (ASM) withdrawal was not statistically different between ASM of 6, 12 or 24 months. In contrast, in persons whose cyst calcified post treatment, longer ASM decreased seizure recurrence. The cumulative incidence ratio (CIR) 1–1.5 years after stopping ASM was 1.79 95% CI: (1.00, 3.20) for patients given 6 versus 24 months treatment.

Anti-inflammatory treatment with corticosteroids in patients treated with ASM compared to patients treated with ASM only showed a statistically significant beneficial effect on seizure reduction (CIR 0.44, 95% CI 0.23, 0.85) and cyst resolution (CIR 1.37, 95%CI: 1.07, 1.75).

Our results indicate that ASM in patients with SEL NCC whose cysts resolved can be withdrawn, while patients whose cysts calcified seem to benefit from prolonged anti-seizure medication. Additional corticosteroid treatment was found to have a beneficial effect both on seizure reduction and cyst resolution.

## Author summary

Neurocysticercosis is an infection of the brain with the tapeworm *Taenia solium*. On the Indian sub-continent, the majority of people suffering from neurocysticercosis only show one cyst (larval form of the parasite) in their brain and often present at clinics with epileptic seizures. There is still some debate on the best way to treat these cases to reduce the risk for seizure recurrence. The goal of this study was to review the literature on how well different durations of treatment with anti-seizure medication (ASM), combined or not with corticosteroids, work in order to reduce the seizure recurrence and eliminate the cyst from the brain. The results show that ASM for 24 months compared to 6 months favors reduced seizure recurrence in patients whose cysts calcified post treatment. Concerning anti-inflammatory treatment, corticosteroids were also found to have beneficial effects on seizure reduction. However, further high-quality randomized controlled trials with adequately long follow-up time are required for better evidence on best drug(s), dose and duration of ASM and corticosteroids for individuals with SEL NCC.

## Introduction

Human neurocysticercosis (NCC) is caused by the zoonotic cestode *Taenia solium* and is responsible for nearly one third of all acquired epilepsy cases in endemic countries, despite being considered as possibly eradicable [1–6]. NCC is also increasingly being diagnosed among travelers and migrants to high-income countries [7–11]. Humans acquire NCC by accidental ingestion of the parasite’s eggs which are released by human *T*. *solium* tapeworm carriers. Once ingested, the eggs hatch in the intestines, and the released oncospheres cross the intestinal mucosa, and migrate through the circulatory system throughout the body until reaching small terminal vessels in tissues, where they form cysticerci. NCC occurs when cysticerci develop in the central nervous system. Symptoms and signs associated with NCC often result from degeneration of the cysticercus and the associated host inflammatory reaction. The most common symptoms in patients diagnosed with NCC are seizures, headaches, focal deficits and signs/symptoms of increased intracranial pressure. A range of other manifestations is described less commonly [12].

In Indian patients, migrants from endemic regions, and pediatric populations, the most common form of NCC found on neuroimaging is a single degenerating brain lesion typically referred to as solitary cysticercus granuloma (SCG) or single enhancing lesion (SEL) [13,14]. In other parts of the world, for example in Latin America, multiple parenchymal NCC is more prominent [15]. These variations of disease manifestation in different geographic regions might be due to complex interactions of host, parasite and environment. Factors such as genetic differences, magnitude of parasite infestation and pork consumption might directly or indirectly impact on disease presentation [15–17].

In individuals with SEL NCC, symptomatic treatment such as anti-seizure medication (ASM) for seizure control and anti-inflammatory drugs for the control of inflammation are commonly used. However, the effectiveness of type, different dosage regimens and duration of ASM use remain controversial. There is also limited evidence to support the time point of ASM withdrawal [18–21]. Albeit not knowing their precise mechanism of action, corticosteroids are believed to have a beneficial effect on seizure outcome, because of their anti-inflammatory and anti-edema properties. [22–25].

In 2016, the World Health Organization (WHO) commissioned the development of “Diagnosis and Treatment Guidelines for *Taenia solium* Neurocysticercosis”. The guidelines aim at improving treatment and diagnosis of parenchymal NCC in resource-poor contexts. The overall objective is to provide guidance on diagnosis and treatment of NCC on the one hand, and on the other hand, to facilitate the implementation of the WHO resolution on epilepsy and neglected tropical diseases (WHA 68.20 and WHA 66.12 respectively). Recommendations for management of people with NCC should be based on firm evidence, part of which the current study has set out to provide, and expert consultations.

The objective of this study was on the one hand, through the systematic review, to estimate the effectiveness of (1) the use of prolonged administration (12–24 months) of ASM compared to shorter regimens (6–12 months) in particular in patients whose SEL did or did not calcify and (2) the use of anti-inflammatory therapy (e.g. prednisolone) in patients with SEL NCC and associated seizure(s)/epilepsy taking ASM on reduction of seizure recurrence and cyst resolution compared to those taking ASM alone and, on the other hand, to add expert knowledge in order to give evidence and experience-based treatment recommendations. The expert knowledge was drawn from the WHO NCC Guideline Development Group (for more detail refer to Methods).

## Methods

### Systematic review/meta-analysis

#### Protocol and registration

The study protocol was developed following PRISMA-P guidelines and conceptualized according to the WHO Guideline Development Proposal (version 28 April 2016) [26]. The review has been registered with PROSPERO (CRD42019087665) [27].

#### Sources, search methods and eligibility criteria

The literature search was designed to identify all studies on treatment of NCC, irrespective of language and time of publication (up to May 2019). Therefore, the following databases were searched: PubMed, EMBASE, Global Index Medicus (limited to Regional Databases LILACS, AIM, WPRIM, IMSEAR, IMEMR), Global Health and Web of Science. Details on the inclusion/exclusion criteria, search term, its adaptation to the respective database and search dates can be found in Supplemental Material S1 and S2 Tables. In addition, the reference lists of included studies and from previous meta-analyses [28–32] were checked for potentially relevant additional publications. Before initiating the systematic review, PubMed, Cochrane and PROSPERO (search term: neurocysticercosis, first date of search: 06/08/2016) were searched for the publication of high-quality systematic reviews or registration of similar studies on this topic in the previous 5 years.

#### Study selection

The identified publications were reviewed in three phases using Covidence [33]. For a decision on including/excluding a publication, two votes from any of the four independent reviewers (JB, AA, PS, CU) were necessary. If opinions were not in agreement and could not be reconciled by referring to the study protocol, a third expert’s opinion was obtained (AW/HG/HC).

In Phase I, titles and abstracts were reviewed. Narrative reviews, studies not involving human subjects and opinion papers were excluded. Studies using experimental or observational study design assessing the effect of treatment of or the validity of diagnostic tests for NCC were included.

In Phase II, publications eligible after Phase I were assessed based on full text. Studies meeting the following inclusion criteria were included at this phase: Patient populations eligible for these two questions were those with a diagnosis of SEL (according to validated criteria) on MRI or CT scan with a well-established diagnosis of epileptic seizures [31,34,35]. The interventions of interest were (1) long ASM (12–24 months’ duration) and (2) anti-inflammatory therapy plus any of the currently marketed ASM. None of the interventions included anti-parasitic treatment. The comparator group for (1) were shorter ASM regimens (6–12 months) and for (2) placebo or ASM alone. The primary outcomes of interest were the cumulative incidence ratios of seizure recurrence and of cyst resolution. The secondary outcome included any adverse events reported by the study authors following drug administration. Only studies with those specific characteristics were eligible for review in Phase III.

In Phase III, the extent to which publications deemed eligible after Phase II addressed the two research questions specific to this systematic review, was determined.

#### Quality assessment

The risk of bias of the included studies was assessed independently by three reviewers (AA, RM and JB), two reviewers for each publication, using the Cochrane risk of bias tool for randomized controlled trials (RCTs) [36]. The confidence in the cumulative evidence was assessed using the GRADE framework and the online tool GRADEpro GDT [37–39]. Funnel plots were used to explore publication bias.

#### Data extraction and items collected

Data extraction was done by three independent reviewers (JB, RM and AA), two of whom were assigned to each paper. Data on general study characteristics, participants, interventions and outcomes was extracted from all studies included at Phase III. The data extraction sheets can be found in S3 Table.

#### Data analysis

Extracted data was synthesized into a narrative review and meta-analyses were performed using RevMan 5.3 when deemed applicable [40]. Heterogeneity among studies was assessed visually through forest plots and statistically with the I^2^ statistic. We used random-effect log-linear models to obtain cumulative incidence ratio estimates with their 95% CI for seizure recurrence due to the heterogeneity in the populations and settings of the different studies and the unreliability of the I^2^ when only a very small number of studies was available. A stratified analysis using random-effect models was also conducted to assess whether calcification of cysts modified the effectiveness of anti-seizure treatment regimens. To test the robustness of our meta-analyses we carried out sensitivity analyses. The confidence in the body of evidence was assessed using the GRADE approach [37–39]. Quality of evidence was graded on the following criteria: risk of bias, inconsistency of results, indirectness of evidence, imprecision and publication bias.

### Expert group-based consensus recommendations

The results of the systematic review were sent out to a panel of experts for their input prior to a personal meeting. The 12 experts were selected from all WHO regions, gender-balanced and with different technical backgrounds including expertise in: epileptology, neurology, neurosurgery, infectious diseases, epidemiology, mental health, zoonotic diseases, parasitology, public health, laboratory experts, programme managers, and health care providers.

During a face-to-face meeting the evidence was discussed and recommendations were given based on consensus and by applying the framework of GRADE [39]. As well as the quality of evidence, additional aspects such as the balance of benefits and harms, values and preferences, feasibility, equity and acceptability, resource requirements and other factors as appropriate were considered. The recommendations were classified as “strong” or “conditional/weak”.

## Results

### Study selection

**Fig 1:**
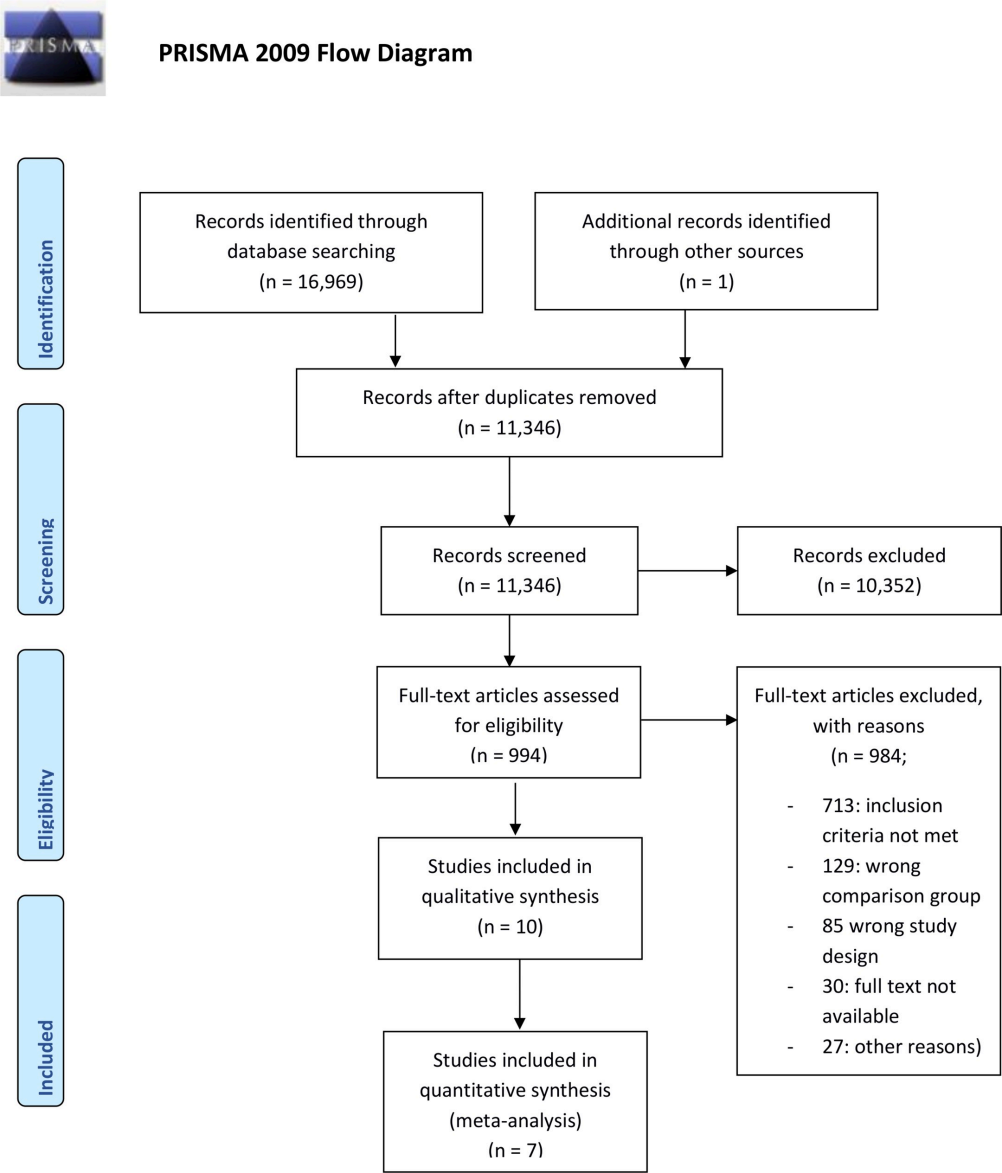
PRISMA flow chart. Presenting the search for relevant studies.

Fig 1 depicts the PRISMA flow diagram of the studies screened [41], excluded and included in the different phases of the review. A total of 16,969 hits were obtained across all search engines, and one additional record was added following review of the references of the included studies. After duplicate removal, 11,346 publications’ titles and abstracts were screened. Following Phase I review, 994 articles were moved to Phase II. During Phase II, 984 articles were excluded due to not meeting the inclusion criteria (713), wrong comparison group (129), wrong study design (85), no access to full text (30) and other reasons (27) leaving 10 eligible publications. Of these eligible papers for qualitative analysis, four studies focused on treatment of SEL NCC with different regimens of anti-epileptic drugs [18–21] and six publications considered different corticosteroid regimens [22–25,42,43] without anti-parasitic drugs. The effect of concomitant anti-parasitic treatment was not within the scope of this research question. An overview of main characteristics of the included studies can be found in S4 Table. Meta-analyses were performed using three and four studies assessing the effectiveness of different regimes of ASM [18,20,21] and corticosteroids [22–25], respectively.

### Effectiveness of different ASM durations on seizure recurrence, lesion resolution, calcification and side effects

Four studies were included to address this research question. However, for the meta-analysis only three of the studies were considered: the study from Singhi et al. was not included further in the quantitative analysis as albendazole was given to 55% of the patients within the first three months without specifying to which group these patients belonged [19]. Albendazole is considered to have a favorable effect on seizure reduction [32,44,45].

### Study and subjects’ characteristics

All included studies were randomized trials, single center studies conducted in northern India [18–21]. Anti-seizure medication duration varied from 6 to 24 months. Seizure recurrence was assessed after tapering off ASM between 12 and 18 months [18–21].The studies were conducted between 1996 and 2004 with two studies not mentioning the study period [19,20]. Only one study reported using some diagnostic criteria for NCC: they reported using the Del Brutto criteria [21].

A total of 496 patients were enrolled in the trials, 237 males, 148 females, and 81 for whom gender was not reported [18]. Thirty patients were lost to follow up, leaving 466 patients with outcome data [19,21]. Patients were aged between 3 and 52 years [18–21].

Only three studies reported on the type of presenting epileptic seizures of patients. Most presented with focal to bilateral tonic-clonic seizures (formerly partial seizures with secondary generalization) (150 patients) or focal aware seizures (formerly simple partial seizures) (143 patients). Focal impaired awareness seizures (formerly complex partial seizures)(56 patients) and generalized seizures (48 patients) were noted less often [19–21,35]. One study only mentioned that most patients presented with focal aware motor seizures with secondary generalization [18]. The same three studies reported on the type of NCC lesions found on neuroimaging. The most common lesion was a ring enhancing lesion (318 patients) with a few presenting with disc enhancing lesions (67 patients) [19–21].

Most of the studies did not report on adverse events, except Verma et al. mentioning not having observed “severe side effects” [21].

### Risk of bias—Anti-seizure medication

**Fig 2:**
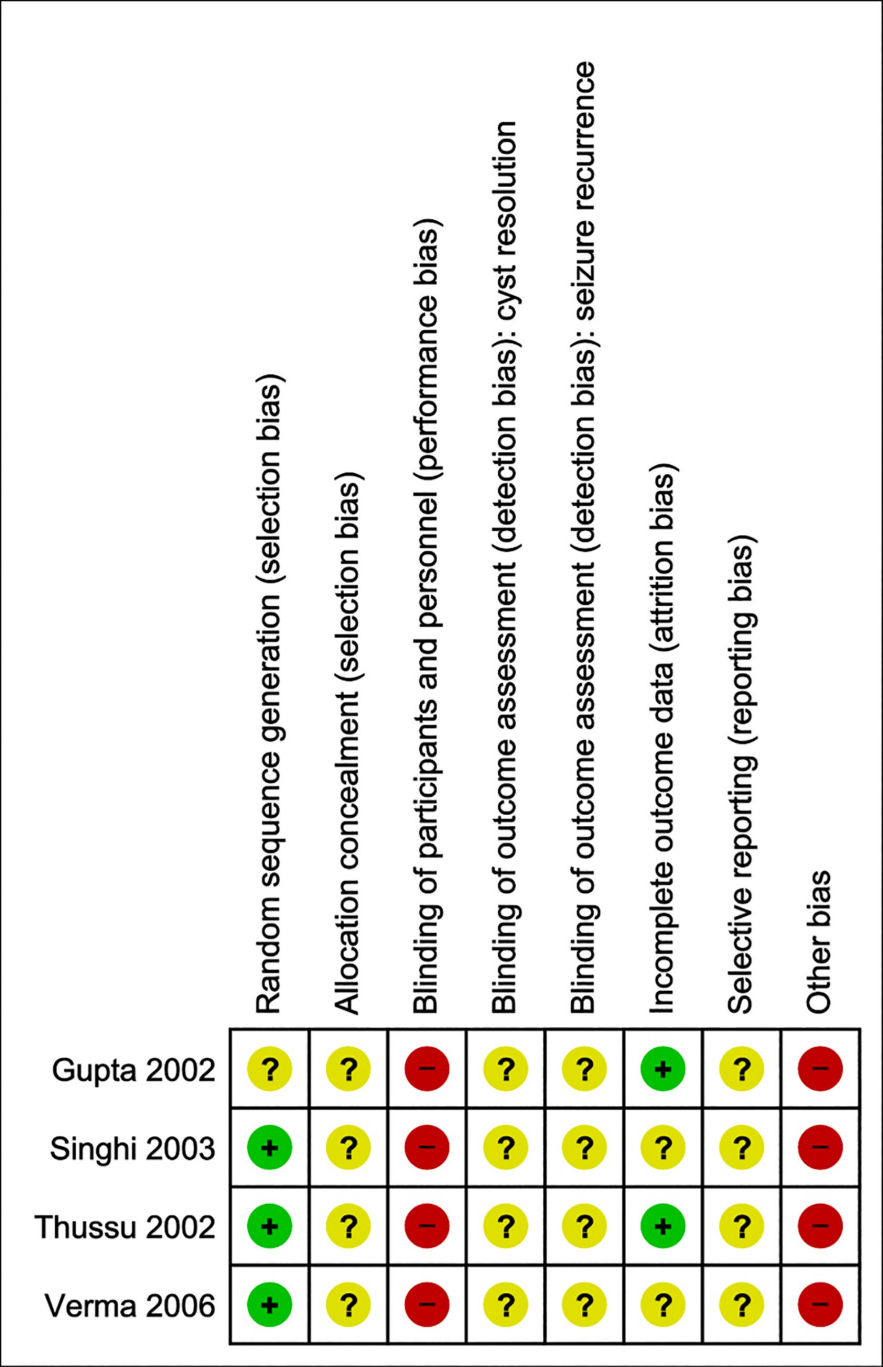
Risk of bias summary—Anti-epileptic drugs. Judgement of the two independent reviewers about each risk of bias item for the included studies.

The results of the Cochrane Risk of Bias assessment are presented in Fig 2. Details of the assessment can be found in S5 Table. Allocation concealment, detection and reporting bias remain unclear, whereas no method of blinding of participants and personnel was applied [18–21]. Most of the studies reported a random component used in the sequence generation (e.g. random number table, coin tossing), thus the risk of selection bias was here considered as low.

Additionally, other concerns for bias included 1) missing information on treatment regimen (drug used and dosage) [18–21] 2) treatment of patients with anti-parasitic drugs (55% of the included patients received albendazole) [19] 3) choice of drugs dependent upon the affordability of drugs by the patients [20] 4) patients with persistence of the lesion were excluded from the study [20,21] 5) different follow up times (Verma et al. followed their patients for at least 18 months after ASM tapering, whereas the other study authors report on at least a year after stopping ASM) [18–21] and 6) none of these studies detailed how adherence to anti-seizure medication was measured and none measured drug levels. As in all studies of ASM, adherence to medication is an important confounder. Further, limited information on study design, methods and results restricts the possibility of further assessing study quality.

### Results of individual studies and meta-analyses for ASM efficacy assessment

**Fig 3:**
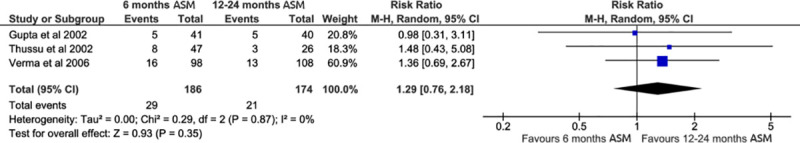
Forest plot. 6 months ASM versus 12–24 months ASM. Outcome: seizure recurrence.

**Fig 4:**
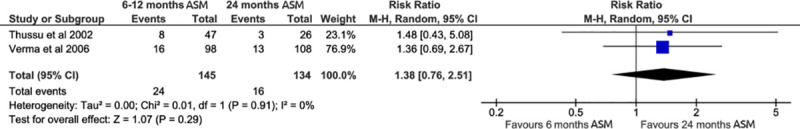
Forest plot. 6 months ASM versus 24 months ASM. Outcome: seizure recurrence.

**Fig 5:**
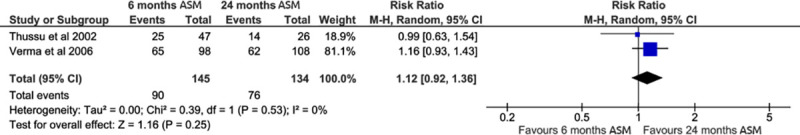
Forest plot. 6 months ASM versus 24 months ASM. Outcome: Cyst resolution.

**Fig 6:**
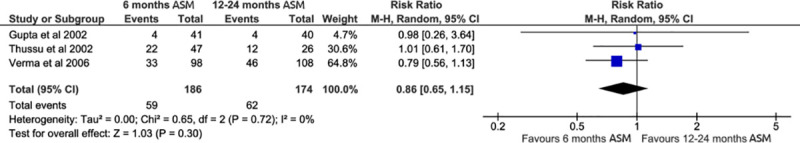
Forest plot. 6 months ASM versus 12–24 months ASM. Outcome: Calcifications.

Fig 3 shows the results of the three studies included in the meta-analysis together with the pooled estimate for the effectiveness of different ASM regimens on the cumulative incidence of seizure recurrence (Figs 3 and 4) cyst resolution (Fig 5) and calcification (Fig 6).

The three studies included 381 patients (176 male, 103 females, 102 unspecified). The most common type of seizures seen in these studies [20,21] were focal aware seizures (119 patients), followed by focal to bilateral tonic-clonic seizures (113 patients), generalized seizures (41 patients) and focal impaired awareness seizures (18 patients). Ring enhancing lesion compared to disc enhancing lesion being the most common radiological lesion (235 patients versus 44 patients) [18,20,21].

Cumulative incidence ratios for 1) 6 months versus 12–24 months and 2) 6–12 months versus 24 months ASM were pooled using a random effects model. No statistically significant effect comparing 6 months to 12–24 months nor 6–12 months versus 24 months treatment could be detected (cumulative incidence ratio (CIR) 1.29, 95% confidence interval (CI): (0.76; 2.18) and CIR 1.38 CI: (0.76; 2.51)). The sensitivity analysis can be found in S1–S18 Figs.

#### Stratified analysis by cyst calcification status

Table 1 below presents the 1–1.5 year cumulative incidence of seizure relapse in patients with and without residual or calcified lesions.

**Table 1:** One-year cumulative incidence of seizure relapse in patients with and without residual or calcified lesions.

Cumulative incidence of seizure relapse in patients		
	with residual or calcified lesions	without residual or calcified lesions
Gupta	NA	NA
Thussu 2002	32.4% (11/34)	0% (0/39)
Verma 2006	30.4% (24/79)	3.9% (5/127)
Singhi 2003*	15.0% (6/40)	0% (0/60)

NA: not available

* In the study of Singhi et al. 2003 three of the patients included in the numerator had calcified lesions and the other three persistence of lesion. For the 40 patients in the denominator it was not further specified. Thusssu and Verma had only participants with calcification in their denominators.

Calcifications were seen in 113 patients, 55 patients in the shorter duration and 58 in the longer treatment duration group (6 months versus 24 months treatment) [18,20,21]. Among the group of patients with a calcified cyst, 22 out of 55 and 13 out of 58 patients (6 months compared to 24 months treatment) experienced seizure recurrence [20,21].

**Fig 7:**
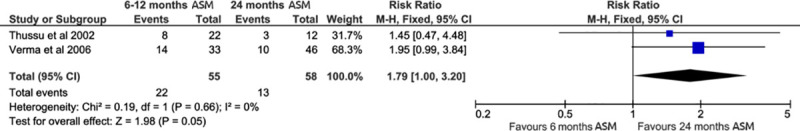
Forest plot of sub-group (patients, where cysts calcified). 6 months ASM versus 24 months ASM. Outcome: seizure recurrence.

A subgroup analysis of seizure recurrence in patients with a calcified lesion reached borderline significance with a pooled CIR of 1.79 CI: (1.00; 3.20) thus favoring 24 months treatment versus 6 months treatment in this particular subgroup (Fig 7).

No further subgroup analysis could be performed due to the limited data.

### Effectiveness of different anti-inflammatory regimens on seizure recurrence, lesion resolution and side effects

Six studies were deemed eligible for the qualitative review while four were kept in the quantitative analyses [22–25,42,43]. Two studies were not further considered: In one study the authors gave methylprednisolone intravenously (all other studies, in contrast, had oral administration) [42]. This study was not further considered as the route of drug administration may influence its bioavailability and therefore could have shown different drug effects. In the second study, corticosteroids were compared to albendazole alone and a combination of albendazole and corticosteroids, so the study design was not comparable with the other included studies [43].

Prakash et al. [42] included 52 patients with new-onset seizures and a “single CT lesion of cysticercus” in their open-label, randomized controlled trial. The patients were either treated intravenously with methylprednisolone for 5 days plus ASM or with ASM alone. The study authors report that patients receiving an anti-inflammatory drug had a higher proportion of patients saw their lesion disappear after a two months follow-up period (60% versus 18.5%) and fewer patients experienced seizure recurrence (16% versus 33%) after 9 months of follow-up.

Singhi et al. [43] included 133 children with focal seizures and single small enhancing CT lesions in their randomized controlled trial to receive either corticosteroids (S), albendazole (A) or corticosteroids and albendazole (SA) for 28 days. Of the initial patients, 23 were lost to follow-up, leaving 38 patients in group S, 37 in group A and 35 in group SA. After a follow up of 3 and 6 months, cysts had resolved in 52.6% and 76.3% (S), 59.5% and 75.7% (A), and 62.9% and 74.2% (SA) of patients upon CT examination, respectively. Seizure recurrence was observed in 23 patients while being treated with ASM of whom 36.8% were in group S, 13.5% in group A and 11.4% in group SA. After ASM withdrawal, seven children, three in group S and two in group A and SA presented with seizure recurrence. Thus the authors concluded that corticosteroids alone are not recommended for management of SEL NCC in children.

#### Study and subjects’ characteristics

The four studies included in the meta-analysis were single center and conducted in northern India, two in Lucknow, one in Chandigarh and one in Varanasi [22–25]. The study period ranged between 2001 and 2008. A total of 416 patients were included, 260 males and 156 females between the ages of 6 years and 36 years [22–25]. For cyst resolution the follow-up varied between 8 weeks and 6 months, and for seizure recurrence from 6 months to 12 months. All four studies were randomized and three of the studies were placebo controlled [22,23,25].

Two of the studies explicitly mentioned diagnosis of SCG based on the diagnostic criteria established by Rajshekhar et al. [22,23], while Mall et al. list the corresponding inclusion criteria [24]. Singla et al. refer to “previously validated criteria for SCG” without further clarification [25].

Most of the patients presented with partial seizures with secondary generalization (233 patients), followed by patients with simple partial seizures (122 patients), others (50 patients). The predominant type of lesion was ring enhancing lesion (378 patients). Disc enhancing lesion was described in 27 patients [22–25]. Eleven patients were not described [24].

Cyst resolution was regarded in all studies as a complete resolution of the lesion with no residual scar, calcification or edema or being reported as “normal” [22–25]. Three of the studies used CT for evaluation [22–24], while Singla et al. used both CT and MRI [25]. Epileptic seizure evaluation was based on eyewitness account and classified according to the International League against Epilepsy classification of epileptic seizure types [22–24]. Singla et al. report on using seizure diaries [25].

Three studies reported on the development of cutaneous adverse events, with Mall et al. reporting erythema multiforme and Steven-Johnson syndrome in five and two patients while under carbamazepine treatment, respectively [22,24,25].

Further characteristics of the included studies can be found in the data extraction sheets in S3 Table.

### Risk of bias—Anti-inflammatory treatment

**Fig 8:**
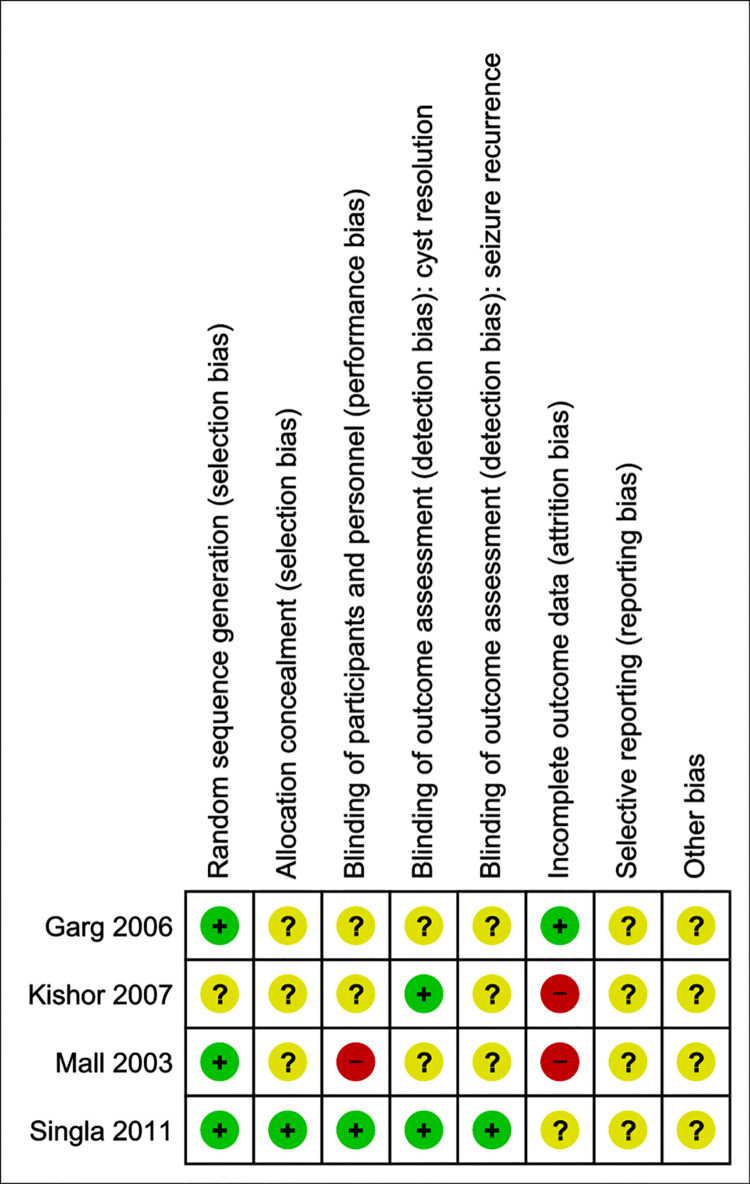
Risk of bias summary—Corticosteroids. Judgement of the two independent reviewers about each risk of bias item for the included studies.

Most of the studies clearly state their method of allocation sequence generation, either using a random number table or a computer random number generator and as such were classified as having a low risk of bias. Other information, however, was lacking: blinding of participants and personnel, outcome assessment as well as selective reporting and other biases largely remained unclear. Two studies were judged to have a high risk of attrition bias as patients were excluded due to loss of follow-up [23,24] (see Fig 8).

### Effect of interventions–anti-inflammatory treatment

**Fig 9:**
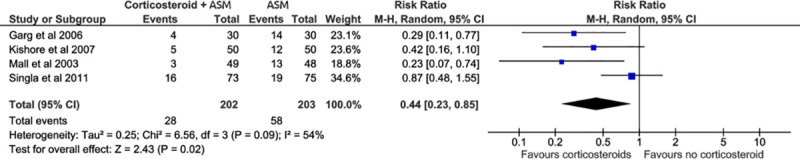
Forest plot of comparison. Corticosteroids versus no corticosteroids. Outcome: seizure recurrence.

**Fig 10:**
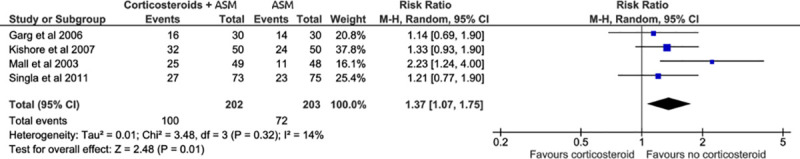
Forest plot of comparison: Corticosteroids versus no corticosteroids. Outcome: cyst resolution without calcification.

Four studies compared oral treatment with corticosteroids to no corticosteroids (Figs 9 and 10) [22–25]: the pooled results indicate a reduction in the 6–12 months cumulative incidence of seizure recurrence in patients receiving corticosteroids compared to those not receiving corticosteroids (CIR 0.44 CI: (0.23; 0.85)) and showed an increase in the 1–6 months cumulative incidence of cyst resolution without calcification (CIR 1.37 CI: (1.07; 1.75)). The sensitivity analysis can be found in S19–S30 Figs.

### Potential biases in the review process

**Fig 11:**
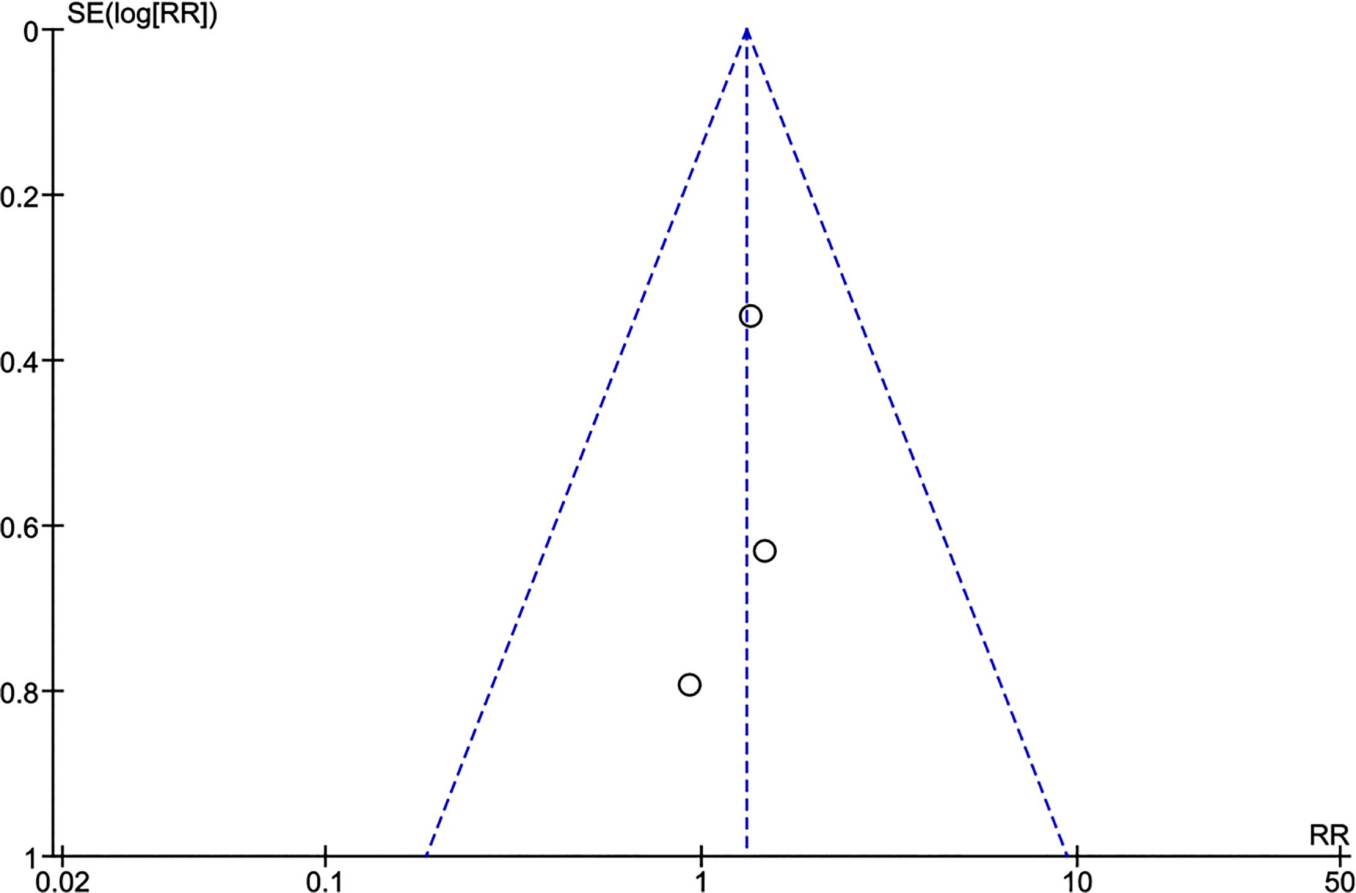
Funnel plot anti-seizure medication. 6 months ASM versus 12–24 months ASM. Outcome: seizure recurrence.

**Fig 12:**
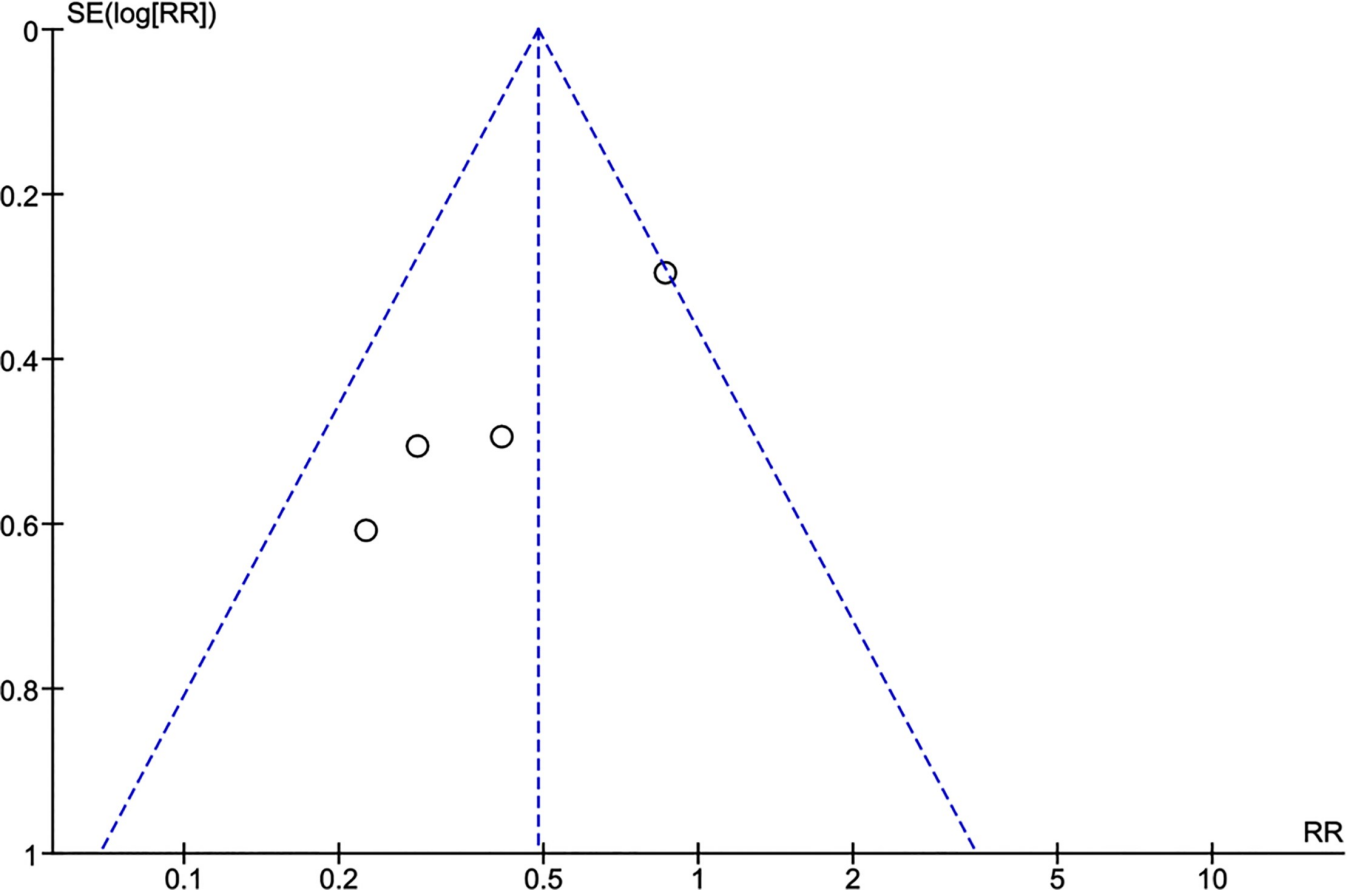
Funnel plot anti-inflammatory treatment. Corticosteroids versus no corticosteroids. Outcome: seizure recurrence.

Figs 11 and 12 depict the funnel plots for the two study questions where no obvious asymmetry was noted visually. Statistical tests were not performed due to the low statistical power due to the small number of included studies. To counterbalance potential publication bias, experts both during the WHO guideline development meeting and previously at a European CYSTINET meeting were asked about personal knowledge of completed or on-going studies. No additional study was identified.

### Summary of findings

For the question on the effectiveness of different ASM duration, the initial high level of evidence due to the study design of RCTs was downgraded because 1) the quality of the included studies was found to yield an overall high risk of bias as described in the above section and 2) differences in populations were found (individuals in whom the cysts calcified versus in whom the cyst resolved) leading to downgrading of the indirectness of the evidence. This resulted in the rating of an overall low quality of evidence meaning that “our confidence in the effect estimate is limited: The true effect may be substantially different from the estimate of the effect” [39]. Considering the sub-group analysis the evidence was rated as moderate (“we are moderately confident in the effect estimate: The true effect is likely to be close to the estimate of the effect, but there is a possibility that it is substantially different”) as the difference in populations was taken into consideration through the sub-group analysis [39] (Table 2).

**Table 2:** GRADE table ASM.

6 or 6–12 months compared to 12–24 or 24 months ASM for individuals with SEL NCC Bibliography: [18–21]											
CERTAINTY ASSESSMENT							SUMMARY OF FINDINGS				
**№ of participants (studies) Follow-up**	**Risk of bias**	**Inconsistency**	**Indirectness**	**Imprecision**	**Publication bias**	**Overall certainty of evidence**	**Study event rates (%)**		**Relative effect (95% CI)**	**Anticipated absolute effects**	
							**With 12–24 or 24 months ASM**	**With 6 or 6–12 months ASM**		**Cumulative incidence with 12–24 or 24 months ASM**	**Cumulative incidence difference with 6 or 6–12 months ASM**
**Seizure recurrence in 6 months compared to 12–24 months A**SM **for individuals with SEL NCC**											
360 (3 RCTs)	serious ^a^	not serious	serious ^b^	not serious	none	⨁⨁◯◯ LOW	21/174 (12.1%)	29/186 (15.6%)	**CIR 1.29** (0.76 to 2.18)	121 per 1.000	**35 more per 1.000** (29 fewer to 142 more)
**Seizure recurrence in 6–12 months compared to 24 months A**SM **for individuals with SEL NCC**											
279 (2 RCTs)	serious^a^	not serious	serious ^b^	not serious	none	⨁⨁◯◯ LOW	16/134 (11.9%)	24/145 (16.6%)	**CIR 1.34** (0.76 to 2.51)	119 per 1.000	**41 more per 1.000** (29 fewer to 180 more)
**Seizure recurrence in 6 months compared to 24 months A**SM **in individuals with SEL NCC, whose cysts had calcified**											
113 (2 RCTs)	serious ^a^	not serious	not serious	not serious	none	⨁⨁⨁◯ MODERATE	13/58 (22.4%)	24/55 (43.6%)	**CIR 2.00** (1.14 to 3.52)	224 per 1.000	**224 more per 1.000** (31 more to 565 more)

**CI:** Confidence interval; **CIR:** Cumulative incidence ratio

Explanations:

a. Overall high risk of bias of included studies

b. differences in populations

For the question on the effectiveness of corticosteroids, the overall body of evidence was rated as low as the indirectness of the evidence was downgraded due to differences in interventions such as treatment durations and drug dosages and the risk of bias was considered as serious (Table 3).

**Table 3:** GRADE table anti-inflammatory treatment.

**Anti-inflammatory treatment plus anti-seizure medication compared to anti-seizure medication alone or with placebo in individuals with SEL NCC Bibliography: [22–25]**											
**CERTAINTY ASSESSMENT**							**SUMMARY OF FINDINGS**				
**№ of participants (studies) Follow-up**	**Risk of bias**	**Inconsistency**	**Indirectness**	**Imprecision**	**Publication bias**	**Overall certainty of evidence**	**Study event rates (%)**		**Relative effect (95% CI)**	**Anticipated absolute effects**	
							**With anti-seizure medication alone or with placebo**	**With anti-inflammatory treatment plus anti-seizure medication**		**Risk with anti-seizure medication alone or with placebo**	**Risk difference with anti-inflammatory treatment plus anti-seizure medication**
**Seizure recurrence (follow up: range 6 months to 12 months)**											
405 (4 RCTs)	serious	not serious	serious ^a^	not serious	none	⨁⨁◯◯ LOW	58/203 (28.6%)	28/202 (13.9%)	**CIR 0.44** (0.23 to 0.85)	286 per 1.000	**160 fewer per 1.000** (220 fewer to 43 fewer)

**CI:** Confidence interval; **CIR:** Cumulative incidence ratio

Explanations:

a. Differences in interventions (applicability)

### Recommendations of the WHO guideline development group

For the ASM of SEL NCC, the experts reached consensus that: 1) ASM therapy withdrawal should be considered after six months in individuals with SEL NCC and epileptic seizures/epilepsy with low risk of seizure recurrence such as patients with a resolved granuloma, no residual calcification and who have been seizure free for at least 3 months, and 2) ASM therapy should be continued if the lesion persists or calcifies after 6 months based on neuroimaging. Patients should be closely monitored while ASM are being withdrawn.

It needs to be noted that SEL NCC can cause both acute symptomatic epileptic seizures and epilepsy. The latter typically occurs once the granuloma has resolved and there is presumably an epileptogenic scar. Persistent granulomas on the other hand can cause repeated acute symptomatic epileptic seizures with the exception that in some persistent granulomas with multiple epileptic seizure episodes there is a possibility that the epileptogenic circuit establishes before the granuloma resolves. Thus, while acute symptomatic epileptic seizures can be treated with short courses of ASM till the granuloma resolves, epilepsy and epileptic seizures with additional risk factors will need long term anti-seizure medication.

The strength of these recommendations was rated as conditional since limited evidence is available. Additionally, the experts emphasized that besides treatment evidence, values and preferences, side effects as well as cost, availability of drugs and human rights issues (e.g. stigmatization, discrimination) should be considered in patient care [39].

For anti-inflammatory treatment, consensus was reached that a combination of anti-parasitic therapy in the form of albendazole with corticosteroids is recommended for the treatment of individuals with symptomatic SEL NCC [45]. Corticosteroids are recommended to be started 24 to 48 hours prior to anti-parasitic therapy. A beneficial effect was also seen for corticosteroids alone. The recommendation was rated by the expert panel as conditional.

## Discussion

The cumulative incidence of seizure recurrence 1–1.5 years after ASM withdrawal was lower among patients treated with ASM for 24 months compared to those treated for 6 months when the cyst calcified following treatment. Such difference was not noted in patients in whom the cyst resolved entirely following treatment. When this effect modification was ignored, no benefit of longer ASM could be noted. The addition of corticosteroids to ASM treatment resulted in lower seizure recurrence and higher cyst resolution post-treatment.

The strength of this work lies in a very exhaustive search (search term and inclusion of databases), a stringent risk of bias assessment and overall assessment of the body of evidence via the GRADE approach. Another strength of the study is the combination of evidence and expert knowledge: the expert group included various recognized international experts from multiple disciplines with longstanding clinical expertise in treating NCC patients from all over the world. However, some limitations remain, which are discussed carefully below:

### Anti-seizure medication

Overall, the studies had methodological limitations such as small sample sizes, lack of blinding, different time points of follow-up imaging, lack of description of study withdrawals and limitations of generalizability as all the studies were conducted in North India. Another limitation is that choice, dose and adherence to ASM was not detailed. Careful measures of adherence such as patient logs, pill counts, and drug levels were not reported and could bias the results substantially. Additionally, as the included studies were not recent, older ASM were used (carbamazepine and phenytoin). New ASM have become available since then, which should be preferred according to the guidelines of the International League against Epilepsy to avoid anticonvulsant hypersensitivity syndrome and the potential interaction with anthelminthics [46–48]. Further studies to evaluate their effectiveness in individuals with SEL NCC are needed.

In addition to all the different aspects of bias evaluated previously, one should also consider that by following up after stopping the treatment, patients with longer ASM schemes benefit not only from the effect of the ASM itself, but also from the effect of time: during a longer time period the natural history of cyst involution can contribute to resolution of the cyst, which then is associated with a reduced probability of seizure recurrence. This could potentially impact the results in the following way: (i) underestimating the effectiveness of longer ASM schemes by neglecting up to 1.5 years of follow-up among those treated for only 6 months (compared to those treated for 24 months), a form of neglected time bias; and (ii) overestimating the effectiveness of longer ASM schemes by ignoring the potential benefit of time on cyst resolution and associated reduction of seizures.

Moreover, to study the recurrence of seizures, for example in patients with a calcified lesion, even a longer follow-up period than reported in the included studies would be required to capture possible events. Since the numbers of seizures (cumulative incidence) increase with time (particularly in those with calcified lesions), a longer duration study arm might possibly have demonstrated the benefits of treating these people with ASM over longer periods of time.

Also, in the studies included in this meta-analysis, children and adults were enrolled. Patients of a younger age group seem to present more often with SEL NCC [49] and drugs should be adapted to this particular patient group.

Another limitation is that mortality and other adverse events (other than seizures after randomization) were not evaluated or reported in any of the studies of this meta-analysis and the socio-psychological dimensions the patients might have to deal with in their daily lives such as stigmatization and economic losses were not further integrated in the context of patient management.

### Anti-inflammatory treatment

The included studies of anti-inflammatory treatment present with methodological problems with limited information on risk of bias assessment; dosage and durations of the drugs used varied, as did follow-up times. Moreover, the follow-up periods were short (up to one year) and do not permit firm conclusions on long-term outcomes. In addition, more detailed information on cyst resolution and calcification in the time course would be of interest.

Another limitation is that all the studies were conducted in India (two of the four studies are from Lucknow) thus raising a generalizability question. One of the two studies conducted outside the center in Lucknow, which is the largest study among those included, showed more inconclusive results regarding the direction of the effect of corticosteroids on seizure recurrence with a larger confidence interval [25].

Most of the studies included children and adults. Only one study included adults (older than 15 years) exclusively [25]. Although exclusion of this study from our sensitivity analysis regarding seizure recurrence did not affect the direction of the results, the question of different effects of corticosteroids (dosage etc.) in children and adults cannot be answered with the available data.

Also, side effects in particular due to long-term steroid use, are not well studied in the included trials.

### Agreement and disagreement with other reviews/studies

#### Anti-seizure medication

A recent meta-analysis by Sharma et al. and Frackowiak et al. evaluated the benefits and harms of different durations of ASM in individuals with SEL NCC [30,50]. The same literature was included in our systematic review as no further study was identified. The study authors found no significant differences, neither when comparison was done between 6 months and 12 to 24 months of therapy (odds ratio (OR) 1.34, CI 95% 0.73 to 2.47) nor between 6 to 12 months and 24 months (OR 1.36, CI 95% 0.72 to 2.57).

We evaluated the results using cumulative incidence ratio as estimator ratios and used random effect models instead of fixed effect models as we believe that the between study heterogeneity is substantial (drugs and dosage not specified etc.). The results of the pooled analysis were similar. However, when considering the included studies all authors pointed out that it is crucial to differentiate between patients in whom cysts calcified and those in whom cysts resolved. Thus, when performing a sub-group analysis of patients whose cysts calcified in our study, anti-seizure medication of 24 months vs 6 months was found to be favoring reduced seizure recurrence, with a statistically significant result.

The expert-based conclusion that ASM should be continued in individuals where the lesion is persisting based on neuroimaging and in those with residual calcification is also supported by a large (185 patients), prospective cohort study with clearly defined criteria and a long follow-up of 2 to 10 years (mean 5.5 years) [51]. The multivariate analysis of the study authors showed that presence of calcific residue on CT scan, occurrence of breakthrough seizures, and occurrence of more than two seizures were risk factors for seizure recurrence. While ASM are withdrawn, patients should be closely monitored.

#### Anti-inflammatory treatment

The use of corticosteroids in the control of inflammation resulting from the natural course of disease or anthelmintic-treatment-induced inflammation remains controversial for SEL NCC. Many experts consider treatment with anthelminthic medication together with corticosteroids as the gold standard. However, as this is not universal clinical practice, the effectiveness of corticosteroids alone as a second level choice treatment option in reducing seizure recurrence is a relevant question.

The arguments against the initial use of anti-parasitic medication for SEL is that after 6 months SEL have started resolving in two thirds of the patients and complete resolution is seen in about 36% according to a large-scale observational study from India [52]. Still, with the use of albendazole and concurrent use of corticosteroids, a small risk of precipitating seizures remains [53]. If the granuloma does not resolve within 6 months, excluding all patients where the natural course of involution cleared the cyst, some experts of the guideline development group argue that albendazole treatment given concomitantly with corticosteroids may hasten resolution of the granuloma in this patient group. However, other experts of the group do recommend the immediate use of anti-parasitic treatment with corticosteroids in patients with SEL and argue that anti-parasitic treatment might lead to better cyst resolution without leaving a calcified residue compared to no treatment, thus decreasing the risk of seizure recurrence. However, there is no clear evidence whether this is the case or not.

Concerning the stand-alone use of corticosteroids different opinions also exist: some experts argue that in the case of SEL, the low-level inflammatory processes may improve the host’s response clearing the granuloma. On the other hand, other experts see a clear benefit in stand-alone corticosteroid therapy as their immunosuppressive properties may reduce the generation of seizure-inducing mediators, control perilesional oedema and decrease damage to neural tissue caused by the inflammatory processes [54,55].

The previously published meta-analyses are not conclusive and arrive at different results using the same included publications, based on differences in the data extraction. In our study we included the same set of studies, however we did not include the study from Prakash et al. in our meta-analysis as they gave intravenous methylprednisolone. We consider this way of administration to have a faster onset of action. That is why we retained this study in the qualitative description of our included studies as it provides valuable information, but not in our meta-analysis.

Our results are in line with the meta-analyses from Cuello-Garcia and Zhao et al. [28,32]: our results suggest a beneficial effect of corticosteroids regarding seizure recurrence. Zhao et al. pointed out that although corticosteroids have a beneficial effect for seizure control and cyst resolution, albendazole and corticosteroids had a greater effect on this outcome [32].

In summary, the quality of the evidence is limited for robust conclusions on effect size and the included studies present various limitations. Thus, further high-quality studies with an adequate sample size and longer follow-up periods would be required to elucidate the role of corticosteroids in the treatment of SEL NCC.

The recommendations given by the guideline development group based on the clinical expertise of the members as well as the evidence presented in this work are in line with the Clinical Practice Guidelines by the Infectious Diseases Society of America and the American Society of Tropical Medicine and Hygiene. Those recommend 1) ASM for all patients with SELs and seizure (strong; moderate), 2) the choice of drugs to be based on availability, side effects, cost and possible interactions with other drugs and 3) tapering off and stopping ASM in patients being seizure free for 6 months with a resolved cystic lesion, with the exception of patients presenting with risk factors for seizure recurrence such as calcifications or residual cystic lesions, breakthrough seizures or with a history of more than two seizures (weak; moderate) [45]. The recommendations regarding anti-inflammatory treatment are congruent with the American guidelines, and differ only in the grading (strong/moderate for the American guidelines).

### Research gaps

Various research gaps on the optimal drug treatment of individuals with SEL NCC exist and further research is necessary. Treatment recommendations should be adjusted when new evidence becomes available.

Concerning ASM of patients with SEL NCC, the following knowledge gaps would require further attention: choice of ASM, monotherapy/polytherapy, dose and duration of anti-seizure therapy, time of withdrawal of ASM and risk factors for seizure recurrence post-treatment. Importantly, an appropriately long duration of follow-up should be chosen, as seizure recurrence can occur several months after resolution of the granuloma. Also, the impact of ASM on the inflammatory process during seizure episodes with its consequence on cyst evolution should be studied further.

Concerning treatment with anti-inflammatory medication in people with SEL NCC, further research on the contribution of corticosteroids to preventing seizure recurrence and cyst resolution is needed. In addition, pharmacokinetics of anti-inflammatory drugs, their mode of action, dosage and length of treatment as well as their efficacy and safety in children with SEL NCC would need to be explored. Further analysis should evaluate the concomitant use of anti-parasitic drugs in individuals with SEL NCC.

## Conclusion

This work shows that clinically it is important to differentiate between patients with SEL NCC whose cysts resolve and those whose cysts calcify requiring different approaches to ASM. Firm evidence is lacking on when best to withdraw ASM. Better assessment of risk factors for seizure recurrence is needed as well as evidence on how long to treat patients with calcifications with ASM. Concerning corticosteroids, they were found to have a beneficial effect on reducing seizure recurrence, albeit with a low certainty of evidence.

## Supporting information

S1 Fig Effect of interventions—Anti-seizure medication. Seizure recurrence 6–12 versus 24 months.
(TIF)

S2 Fig Effect of interventions—Anti-seizure medication. Seizure recurrence 6–12 versus 24 months: sensitivity analysis.
(TIF)

S3 Fig Effect of interventions—Anti-seizure medication. Seizure recurrence 6–12 versus 24 months: sensitivity analysis.
(TIF)

S4 Fig Effect of interventions—Anti-seizure medication. Seizure recurrence 6 versus 12–24 months.
(TIF)

S5 Fig Effect of interventions—Anti-seizure medication. Seizure recurrence 6 versus 12–24 months: sensitivity analysis.
(TIF)

S6 Fig Effect of interventions—Anti-seizure medication. Seizure recurrence 6 versus 12–24 months: sensitivity analysis.
(TIF)

S7 Fig Effect of interventions—Anti-seizure medication. Seizure recurrence 6 versus 12–24 months: sensitivity analysis.
(TIF)

S8 Fig Effect of interventions—Anti-seizure medication. Seizure recurrence 6–12 versus 24 months subgroup analysis.
(TIF)

S9 Fig Effect of interventions—Anti-seizure medication. Seizure recurrence 6–12 versus 24 months subgroup analysis: non-event.
(TIF)

S10 Fig Effect of interventions—Anti-seizure medication. Seizure recurrence 6–12 versus 24 months subgroup analysis: sensitivity analysis.
(TIF)

S11 Fig Effect of interventions—Anti-seizure medication. Seizure recurrence 6–12 versus 24 months subgroup analysis: sensitivity analysis.
(TIF)

S12 Fig Effect of interventions—Anti-seizure medication. Cyst resolution 6 versus 24 months.
(TIF)

S13 Fig Effect of interventions—Anti-seizure medication. Cyst resolution 6 versus 24 months: sensitivity analysis.
(TIF)

S14 Fig Effect of interventions—Anti-seizure medication. Cyst resolution 6 versus 24 months: sensitivity analysis.
(TIF)

S15 Fig Effect of interventions—Anti-seizure medication. Calcification 6 versus 12–24 months.
(TIF)

S16 Fig Effect of interventions—Anti-seizure medication. Calcification 6 versus 12–24 months: sensitivity analysis.
(TIF)

S17 Fig Effect of interventions—Anti-seizure medication. Calcification 6 versus 12–24 months: sensitivity analysis.
(TIF)

S18 Fig Effect of interventions—Anti-seizure medication. Calcification 6 versus 12–24 months: sensitivity analysis.
(TIF)

S19 Fig Effect of interventions—Anti-inflammatory treatment. Seizure recurrence
(TIF)

S20 Fig Effect of interventions—Anti-inflammatory treatment. Seizure recurrence: sensitivity analysis.
(TIF)

S21 Fig Effect of interventions—Anti-inflammatory treatment. Seizure recurrence: sensitivity analysis.
(TIF)

S22 Fig Effect of interventions—Anti-inflammatory treatment. Seizure recurrence: sensitivity analysis.
(TIF)

S23 Fig Effect of interventions—Anti-inflammatory treatment. Seizure recurrence: sensitivity analysis.
(TIF)

S24 Fig Effect of interventions—Anti-inflammatory treatment. Cyst resolution -shorter follow-up time.
(TIF)

S25 Fig Effect of interventions—Anti-inflammatory treatment. Cyst resolution.
(TIF)

S26 Fig Effect of interventions—Anti-inflammatory treatment. Cyst resolution: sensitivity analysis.
(TIF)

S27 Fig Effect of interventions—Anti-inflammatory treatment. Cyst resolution: sensitivity analysis.
(TIF)

S28 Fig Effect of interventions—Anti-inflammatory treatment. Cyst resolution: sensitivity analysis.
(TIF)

S29 Fig Effect of interventions—Anti-inflammatory treatment. Cyst resolution: sensitivity analysis.
(TIF)

S30 Fig Effect of interventions—Anti-inflammatory treatment. Cyst resolution: sensitivity analysis.
(TIF)

S1 Table PICO questions and inclusion/exclusion criteria. Definition of inclusion and exclusion criteria.
(DOCX)

S2 Table Search terms. Search terms with adaptation to specific database; date of search.
(DOCX)

S3 Table Characteristics of included studies. Data extraction sheets.
(DOCX)

S4 Table Overview of main characteristics of included studies. Summary of included studies.
(DOCX)

S5 Table Risk of bias assessment. Descriptions of the judgments made for the included studies.
(DOCX)
