# Supplementary figures and images for: The effectiveness of anti-inflammatory and anti-seizure medication for individuals with single enhancing lesion neurocysticercosis: A meta-analysis and expert group-based consensus recommendations
Source: PLoS Negl Trop Dis. 2021 Mar 31;15(3):e0009193. doi: 10.1371/journal.pntd.0009193 (PMC8057605; doi:10.1371/journal.pntd.0009193)

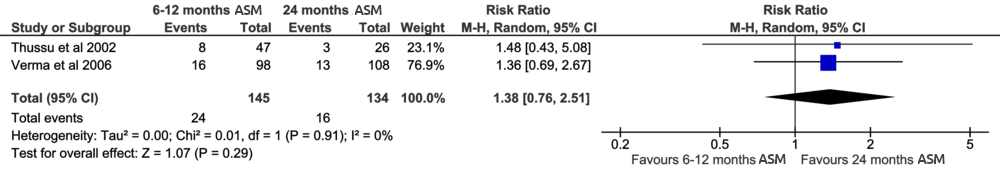

Supplement: S1 Fig — Seizure recurrence 6–12 versus 24 months. (TIF) [file pntd.0009193.s001.tif]

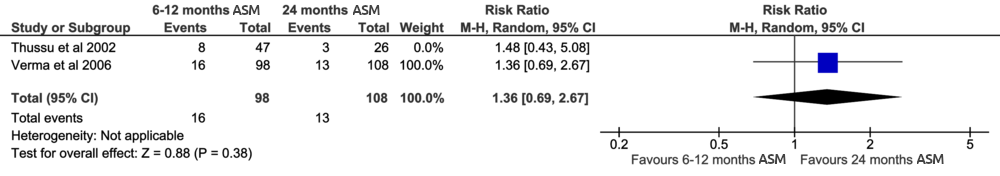

Supplement: S2 Fig — Seizure recurrence 6–12 versus 24 months: sensitivity analysis. (TIF) [file pntd.0009193.s002.tif]

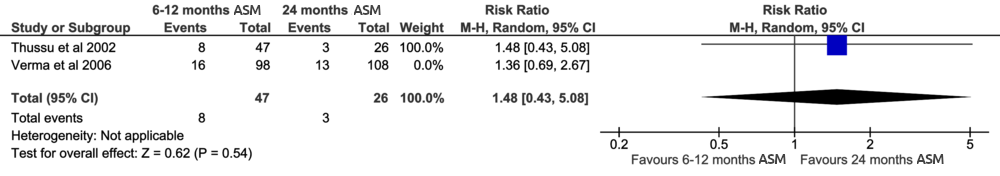

Supplement: S3 Fig — Seizure recurrence 6–12 versus 24 months: sensitivity analysis. (TIF) [file pntd.0009193.s003.tif]

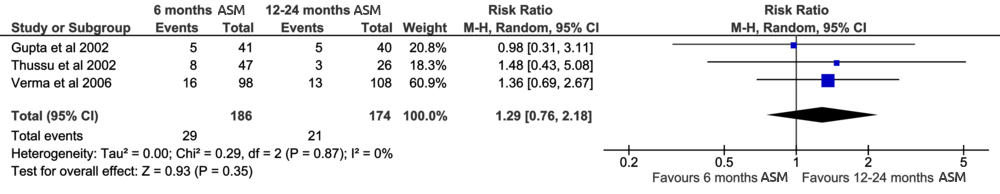

Supplement: S4 Fig — Seizure recurrence 6 versus 12–24 months. (TIF) [file pntd.0009193.s004.tif]

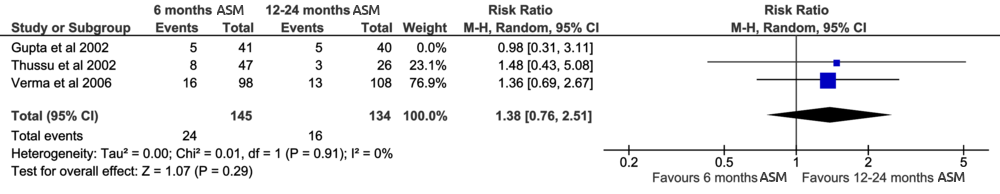

Supplement: S5 Fig — Seizure recurrence 6 versus 12–24 months: sensitivity analysis. (TIF) [file pntd.0009193.s005.tif]

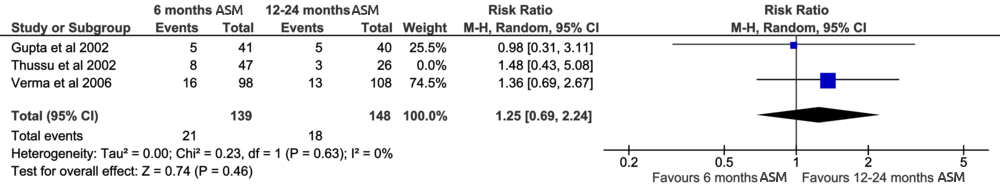

Supplement: S6 Fig — Seizure recurrence 6 versus 12–24 months: sensitivity analysis. (TIF) [file pntd.0009193.s006.tif]

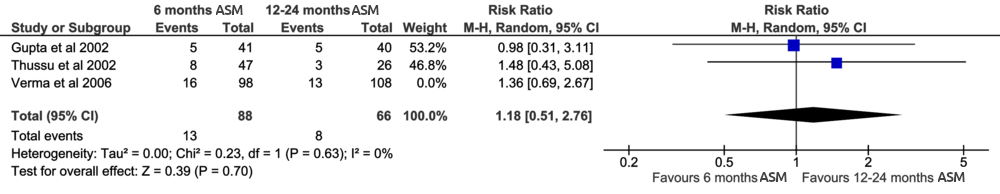

Supplement: S7 Fig — Seizure recurrence 6 versus 12–24 months: sensitivity analysis. (TIF) [file pntd.0009193.s007.tif]

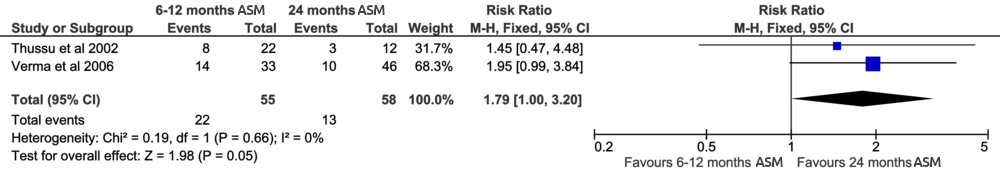

Supplement: S8 Fig — Seizure recurrence 6–12 versus 24 months subgroup analysis. (TIF) [file pntd.0009193.s008.tif]

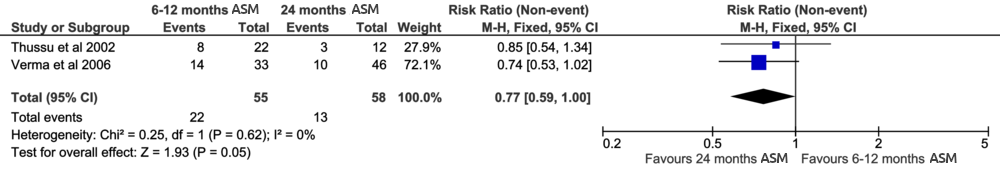

Supplement: S9 Fig — Seizure recurrence 6–12 versus 24 months subgroup analysis: non-event. (TIF) [file pntd.0009193.s009.tif]

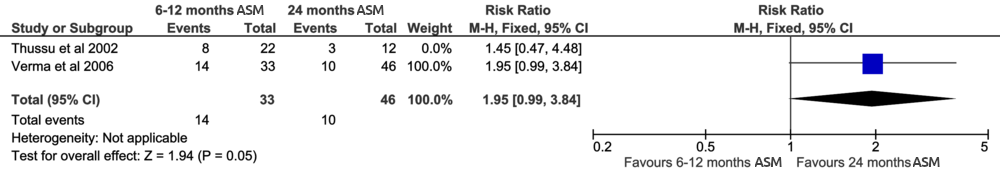

Supplement: S10 Fig — Seizure recurrence 6–12 versus 24 months subgroup analysis: sensitivity analysis. (TIF) [file pntd.0009193.s010.tif]

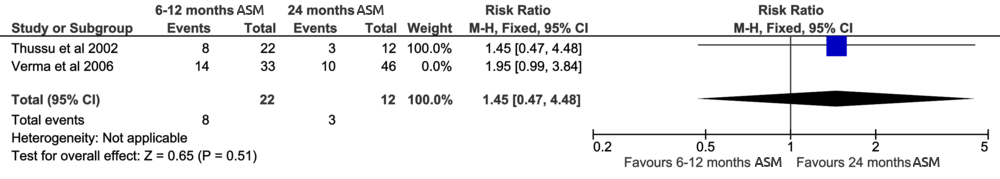

Supplement: S11 Fig — Seizure recurrence 6–12 versus 24 months subgroup analysis: sensitivity analysis. (TIF) [file pntd.0009193.s011.tif]

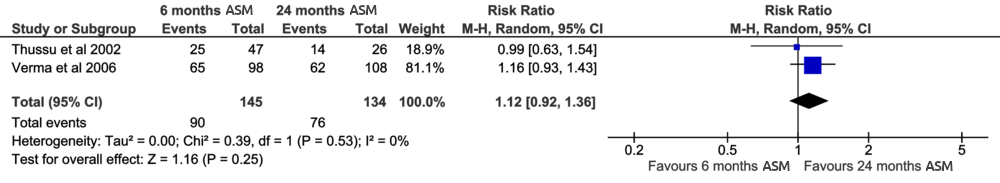

Supplement: S12 Fig — Cyst resolution 6 versus 24 months. (TIF) [file pntd.0009193.s012.tif]

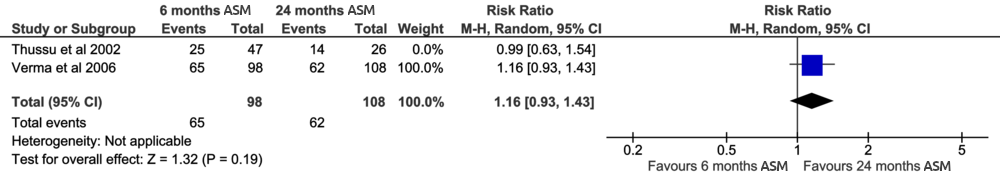

Supplement: S13 Fig — Cyst resolution 6 versus 24 months: sensitivity analysis. (TIF) [file pntd.0009193.s013.tif]

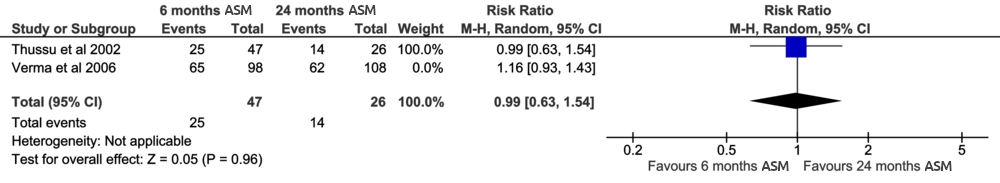

Supplement: S14 Fig — Cyst resolution 6 versus 24 months: sensitivity analysis. (TIF) [file pntd.0009193.s014.tif]

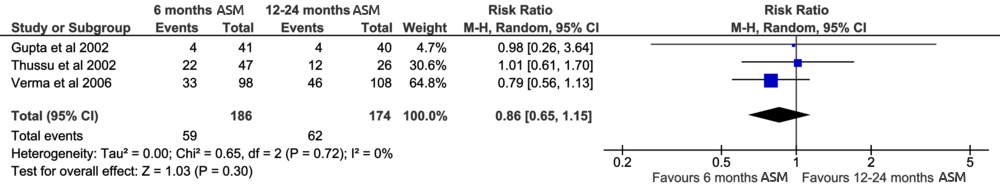

Supplement: S15 Fig — Calcification 6 versus 12–24 months. (TIF) [file pntd.0009193.s015.tif]

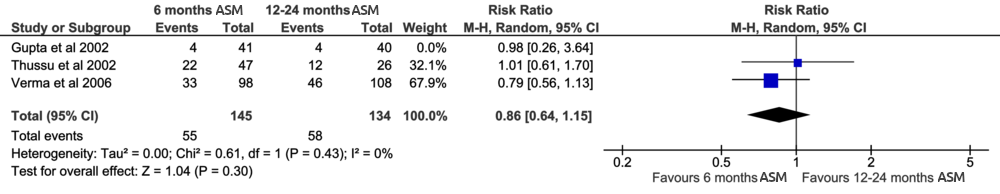

Supplement: S16 Fig — Calcification 6 versus 12–24 months: sensitivity analysis. (TIF) [file pntd.0009193.s016.tif]

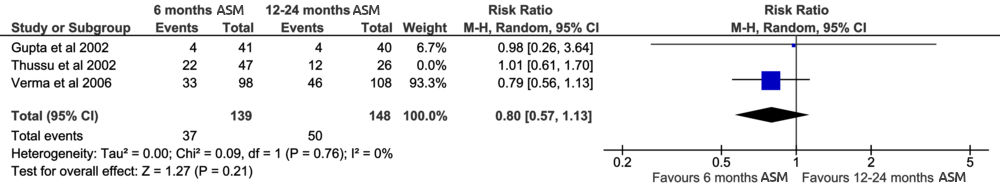

Supplement: S17 Fig — Calcification 6 versus 12–24 months: sensitivity analysis. (TIF) [file pntd.0009193.s017.tif]

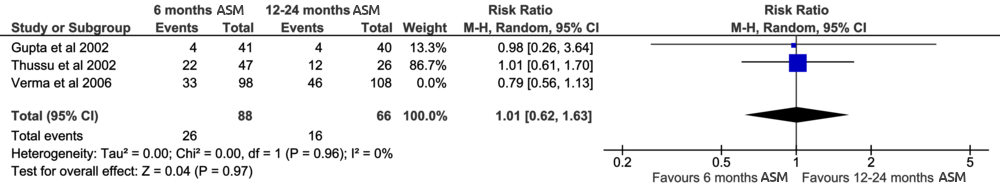

Supplement: S18 Fig — Calcification 6 versus 12–24 months: sensitivity analysis. (TIF) [file pntd.0009193.s018.tif]

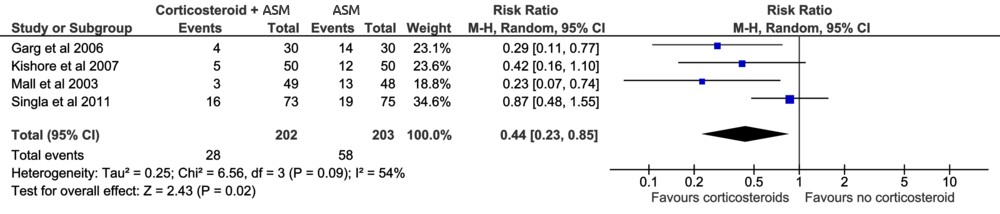

Supplement: S19 Fig — Seizure recurrence (TIF) [file pntd.0009193.s019.tif]

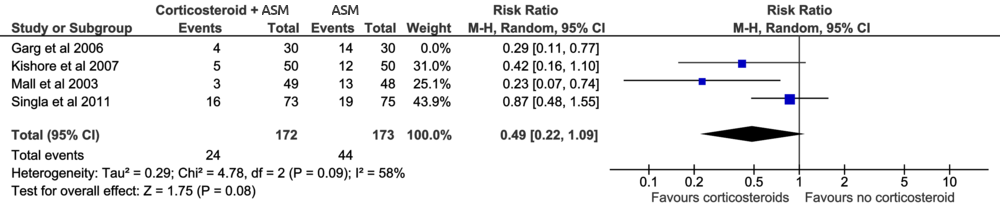

Supplement: S20 Fig — Seizure recurrence: sensitivity analysis. (TIF) [file pntd.0009193.s020.tif]

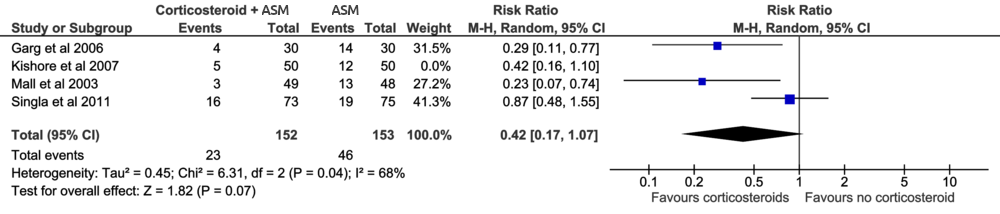

Supplement: S21 Fig — Seizure recurrence: sensitivity analysis. (TIF) [file pntd.0009193.s021.tif]

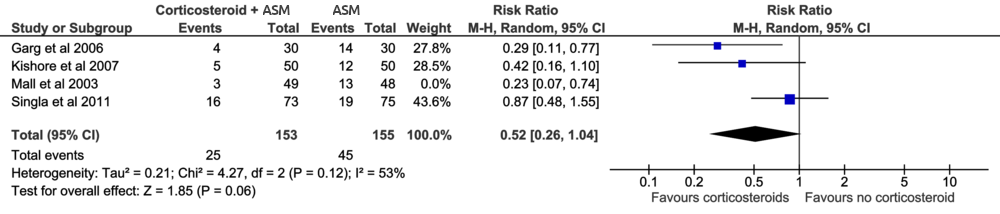

Supplement: S22 Fig — Seizure recurrence: sensitivity analysis. (TIF) [file pntd.0009193.s022.tif]

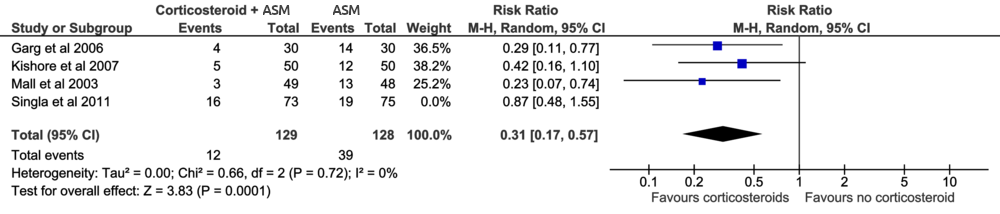

Supplement: S23 Fig — Seizure recurrence: sensitivity analysis. (TIF) [file pntd.0009193.s023.tif]

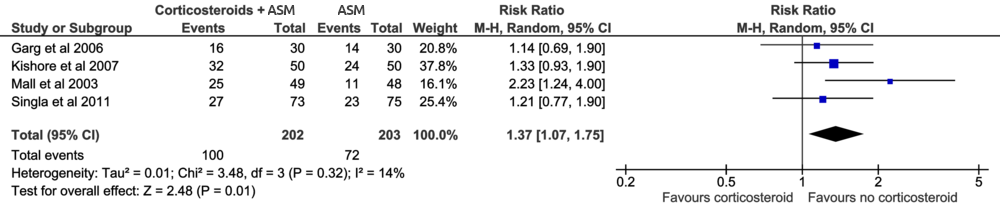

Supplement: S24 Fig — Cyst resolution -shorter follow-up time. (TIF) [file pntd.0009193.s024.tif]

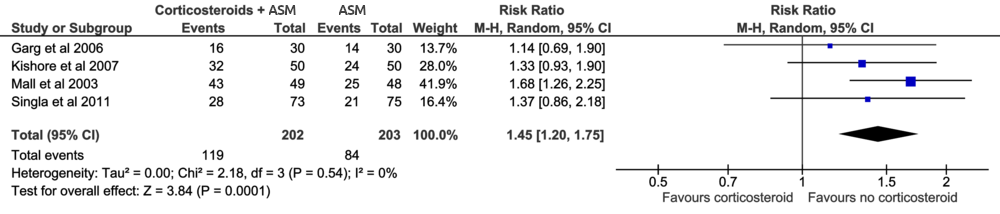

Supplement: S25 Fig — Cyst resolution. (TIF) [file pntd.0009193.s025.tif]

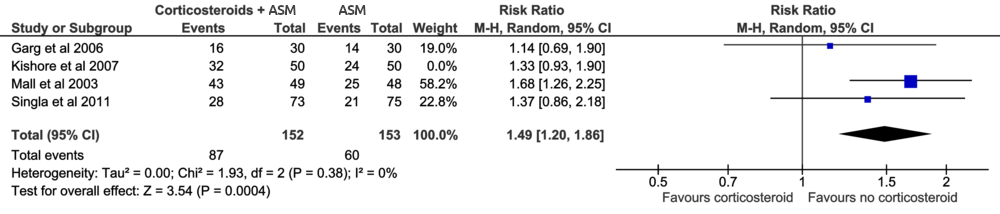

Supplement: S26 Fig — Cyst resolution: sensitivity analysis. (TIF) [file pntd.0009193.s026.tif]

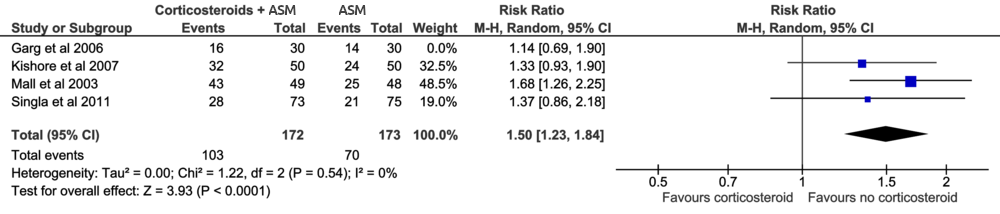

Supplement: S27 Fig — Cyst resolution: sensitivity analysis. (TIF) [file pntd.0009193.s027.tif]

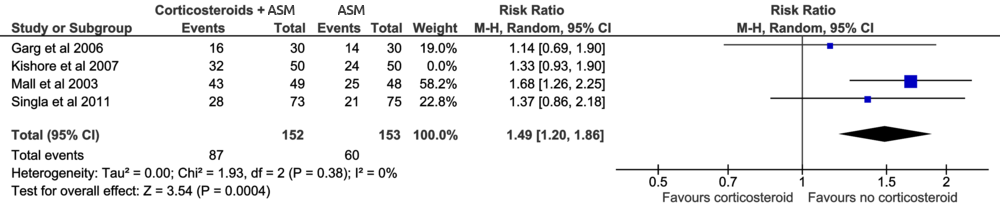

Supplement: S28 Fig — Cyst resolution: sensitivity analysis. (TIF) [file pntd.0009193.s028.tif]

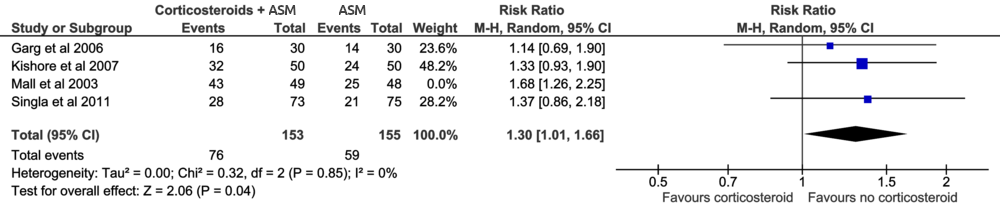

Supplement: S29 Fig — Cyst resolution: sensitivity analysis. (TIF) [file pntd.0009193.s029.tif]

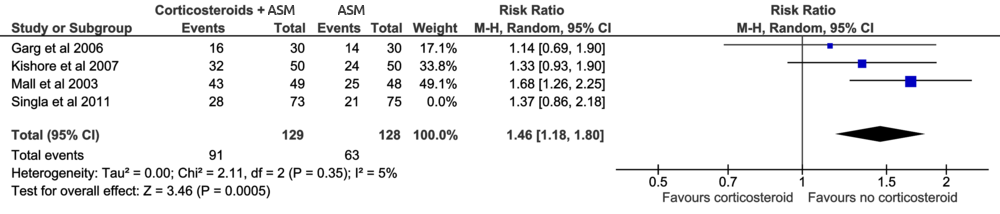

Supplement: S30 Fig — Cyst resolution: sensitivity analysis. (TIF) [file pntd.0009193.s030.tif]
