# Supplementary material for: The effectiveness of anti-inflammatory and anti-seizure medication for individuals with single enhancing lesion neurocysticercosis: A meta-analysis and expert group-based consensus recommendations
Source: PLoS Negl Trop Dis. 2021 Mar 31;15(3):e0009193. doi: 10.1371/journal.pntd.0009193 (PMC8057605; doi:10.1371/journal.pntd.0009193)
Supplement: S1 Table — Definition of inclusion and exclusion criteria. (DOCX) [file pntd.0009193.s031.docx]

**S1 Table. PICO questions and inclusion/exclusion criteria.** Definition of inclusion and exclusion criteria.

| **PICO QUESTION:1** | |
| --- | --- |
| Population | individuals with single enhancing lesion NCC and associated seizure(s)/epilepsy |
| Intervention | prolonged administration (12-24 months) of AEDs |
| Comparator | shorter regimes of AEDs (6-12 months) |
| Outcome | reduction in seizure recurrence (incidence rate) |

| **Inclusion and exclusion criteria** | |
| --- | --- |
| Inclusion criteria | Types of studies:  Experimental studies and observational studies.  Types of participants:  Individuals with *T. solium* single enhancing lesion NCC on MRI or CT scan with well-established diagnosis of epilepsy.  Types of intervention:  The intervention group and control group may have received any of the currently marketed AEDs.  Types of outcome measures:  Seizure recurrence over a specific period of time. |
| Exclusion criteria | Case series and case reports were excluded. *T. solium* NCC patients other than with single enhancing lesions were also excluded |

| **PICO QUESTION: 2** | |
| --- | --- |
| Population | individuals with symptomatic NCC with single enhancing lesion |
| Intervention | anti-inflammatory therapy and anti-epileptic treatment |
| Comparator | anti-epileptic treatment alone |
| Outcome | reduction in seizure recurrence (Incidence rate) |

| **Inclusion and exclusion criteria** | |
| --- | --- |
| Inclusion criteria | Types of studies:  Experimental studies and observational studies.  Types of participants:  Individuals with *T. solium* single enhancing lesion NCC on MRI or CT scan with well-established diagnosis of epilepsy.  Types of intervention:  The intervention group may have received any of the marketed anti-inflammatory therapies and received any of the currently marketed AEDs. The control group may have received any of the currently marketed AEDs  Types of outcome measures:  Seizure recurrence over a specific period of time. |
| Exclusion criteria | Case series and case reports were excluded. *T. solium* NCC patients other than with single enhancing lesions were also excluded |
