# Supplementary material for: The effectiveness of anti-inflammatory and anti-seizure medication for individuals with single enhancing lesion neurocysticercosis: A meta-analysis and expert group-based consensus recommendations
Source: PLoS Negl Trop Dis. 2021 Mar 31;15(3):e0009193. doi: 10.1371/journal.pntd.0009193 (PMC8057605; doi:10.1371/journal.pntd.0009193)
Supplement: S2 Table — Search terms with adaptation to specific database; date of search. (DOCX) [file pntd.0009193.s032.docx]

**S2 Table. Search terms.** Search terms with adaptation to specific database; date of search.

| **#** | **Searches** | **Results** |
| --- | --- | --- |
|  | **PubMed ([http://www.pubmed.gov](http://www.pubmed.gov/))** |  |
| **1** | ("Neurocysticercosis"[Mesh] OR Neurocysticercos* [TW] ) OR ((brain[TW] OR cerebral [TW] OR “Central Nervous System”[ TW] OR “Central Nervous”[TW] OR CNS[TW]OR neuro[TW] OR intramedullary[TW] OR extramedullary[TW] OR medullary[TW] OR ventricular[TW] OR subarachnoid*[TW] OR spinal[TW] OR intraparenchymal[TW] OR extraparenchymal[TW] OR parenchymal[TW] OR intraventricular[TW] OR subarachnoid[TW] OR calcified[TW] OR viable[TW] OR “single enhancing”[TW] OR active[TW] OR inactive[TW] OR headache*[TW] OR “intracranial hypertension” [TW] OR “neurological symptoms” [TW] OR epilepsy [TW] OR “seizures” [MH] OR seizure* [TW] OR “intracranial pressure”[TW]) AND (CYSTICERC* [TW] OR “CYSTICERCOSIS” [MH] OR “brain cysts” [TW] OR “brain cyst” [TW] )) NOT ("Animals" [MH] NOT (HUMANS [MH] AND Animals [MH])) | **4121** |
|  | **EMBASE ([http://www.embase.com](http://www.embase.com/))** |  |
| **2** | 'neurocysticercosis'/exp OR Neurocysticercos*:ti,ab,de OR ((brain:ti,ab,de OR cerebral:ti,ab,de OR ‘Central Nervous‘:ti,ab,de OR CNS:ti,ab,de OR neuro:ti,ab,de OR intramedullary:ti,ab,de OR extramedullary:ti,ab,de OR medullary:ti,ab,de OR ventricular:ti,ab,de OR subarachnoid*:ti,ab,de OR spinal:ti,ab,de OR intraparenchymal:ti,ab,de OR extraparenchymal:ti,ab,de OR parenchymal:ti,ab,de OR intraventricular:ti,ab,de OR subarachnoid:ti,ab,de OR calcified:ti,ab,de OR viable:ti,ab,de OR ‘single enhancing ‘:ti,ab,de OR active:ti,ab,de OR inactive:ti,ab,de OR headache*:ti,ab,de OR ‘intracranial hypertension ‘:ti,ab,de OR ‘neurological symptoms‘:ti,ab,de OR epilepsy:ti,ab,de OR 'seizure'/exp OR seizure*:ti,ab,de OR ‘intracranial pressure ‘:ti,de,ab) AND (CYSTICERC*:ti,ab,de OR 'cysticercosis'/exp OR ‘brain cysts‘:ti,ab,de OR ‘brain cyst‘:ti,ab,de)) AND embase/lim | **7239** |
|  | **Global Index Medicus <http://www.globalhealthlibrary.net/> limited to Regional Databases LILACS, AIM, WPRIM; IMSEAR, IMEMR** |  |
| **3** | (mh:(Neurocysticercosis)) OR Neurocysticercos* OR neurocisticercos* OR ((brain OR cerebral OR (Central Nervous) OR CNS OR neuro OR intramedullary OR extramedullary OR medullary OR ventricular OR subarachnoid* OR spinal OR intraparenchymal OR extraparenchymal OR parenchymal OR intraventricular OR subarachnoid OR calcified OR viable OR (single enhancing) OR active OR inactive OR headache* OR (intracranial hypertension) OR (neurological symptoms) OR epilepsy OR seizure* OR (intracranial pressure)) AND (CYSTICERC* OR “brain cysts” OR (brain cyst))) | **1394** |
|  | **Global Health (CABI) <https://www.cabdirect.org/>** |  |
| **4** | neurocysticercosis OR (((brain OR cerebral OR "Central Nervous System" OR "Central Nervous" OR CNSOR neuro OR intramedullary OR extramedullary OR medullary OR ventricular OR subarachnoid* OR spinal OR intraparenchymal OR extraparenchymal OR parenchymal OR intraventricular OR subarachnoid OR calcified OR viable OR "single enhancing" OR active OR inactive OR headache* OR "intracranial hypertension" OR "neurological symptoms" OR epilepsy OR seizure* OR "intracranial pressure") AND (CYSTICERC* OR "brain cysts" OR "brain cyst" ))) | **2322** |
|  | **Web of Science (Emerging Sources Citation Index (ESCI) --2015-present) <http://apps.webofknowledge.com/>** |  |
| **5** | neurocysticercosis OR (((brain OR cerebral OR "Central Nervous System" OR "Central Nervous" OR CNSOR neuro OR intramedullary OR extramedullary OR medullary OR ventricular OR subarachnoid* OR spinal OR intraparenchymal OR extraparenchymal OR parenchymal OR intraventricular OR subarachnoid OR calcified OR viable OR "single enhancing" OR active OR inactive OR headache* OR "intracranial hypertension" OR "neurological symptoms" OR epilepsy OR seizure* OR "intracranial pressure") AND (CYSTICERC* OR "brain cysts" OR "brain cyst" ))) | **28** |

**Total of publications identified:** 13 777

**Wishor Method applied (Deduplication):** Trash 3786 references

**Date of search:** 20.11.2016

**Up-date of search:** 02.05.2019

**Search terms developed:** by Javier Bustos and Annette Abraham

with supervision by Hector H. Garcia and Andrea S. Winkler

**Search conducted:** by Tomas Allen (World Health Organization)
