# Supplementary material for: The effectiveness of anti-inflammatory and anti-seizure medication for individuals with single enhancing lesion neurocysticercosis: A meta-analysis and expert group-based consensus recommendations
Source: PLoS Negl Trop Dis. 2021 Mar 31;15(3):e0009193. doi: 10.1371/journal.pntd.0009193 (PMC8057605; doi:10.1371/journal.pntd.0009193)
Supplement: S3 Table — Data extraction sheets. (DOCX) [file pntd.0009193.s033.docx]

**S3 Table. Characteristics of included studies.** Data extraction sheets.

**PICO 1**

| **Gupta et al. 2002** **(1)** | |
| --- | --- |
| Study characteristics | India - Department of Neurology, G.B. Pant Hospital, New Delhi  Randomized trial  Duration: January 1996 to July 1999 |
| Participants | Diagnostic criteria: NA  Follow up: 1 year after stopping AED  Repeat imaging of the brain (CT/MRI) was done at 3 months and/or 6 months after imaging  Number of patients: 81, Male: NA  Group A: 41; Group B: 40;  Patient’s characteristic: NA  Lost to follow-up: 0  Seizure frequency: the mean number of seizures was 2.3 in group A and 2.5 in group B |
|  | **Inclusion criteria:**  “All patients of epilepsy with small single enhancing CT scan/MRI brain lesion were included in the study. The diagnosis was based on CT findings of small (< 20 mm) single parenchymal brain lesion surrounded by edema and ring or disc enhancement in contrast CT scan.”  **Exclusion criteria:**  “Patients with suspected tuberculoma or those treated with albendazole were excluded from the  Study.” |
| Interventions | **Group A:**  AED: NA  Treatment duration: 6 months  **Group B:**  AED: NA  Treatment duration: 12 months |
| Outcomes | **Seizure recurrence:** Group A: 5 patients (after 4.3+1.52 months of stopping treatment) Group B: 5 patients (after 2.2+1.48 months of stopping treatment) **Calcifications:** Group A: 4 patients (4 out of the 5 patients with seizure recurrence calcifications) Group B: 4 patients (4 out the 5 patients with seizure recurrence calcifications) |

| **Thussu et al. 2002** **(2)** | |
| --- | --- |
| Study characteristics | India - Department of Neurology, Postgraduate Institute of Medical Education and Research, Chandigarh  Randomized trial  Duration: NA |
| Participants | Diagnostic criteria: NA  Follow up: 1 year after stopping AED, two monthly  Number of patients: 73, Male: 39  Group A: 47; Group B: 26;  Patient’s characteristic: Children and adults  Lost to follow-up: 0  Seizure frequency: NA |
|  | **Inclusion criteria:**  SSECTL on contrast CT scan (ring enchancing/disc lesion < 20mm in size)  **Exclusion criteria:**  “cases who had persistence of the lesion, were put on albendazole and excluded from the study.” |
| Interventions | **Group A:**  AED: “25 patients were treated with carbamazepine and 22 with phenytoin. The choice of drugs depended upon the affordability of drugs by the patients”  Treatment duration: “6 months and then tapered over next three months”  **Group B:**  AED: “13 patients were put on carbamazepine and 13 were on phenytoin”  Treatment duration: 24 months and “then drugs were tapered off over the next 3 months. Patients were followed up for one year after withdrawal of antiepileptic therapy for possible recurrence” |
| Outcomes | **Seizure recurrence:** Group A: 8 patients Group B: 3 patients **Calcifications:** Group A: 22 patients  Group B: 12 patients  **Complete cyst resolution:**  Group A: 25 patients  Group B: 14 after 3 months CT |

| **Verma et al. 2006** **(3)** | |
| --- | --- |
| Study characteristics | India - Department of Neurology, Institute of Medical Sciences, Banaras Hindu University, Varanasi  Randomized trial  Duration: March 1999 – December 2004 |
| Participants | Diagnostic criteria: Del Brutto revised criteria  Follow up: “8 weeks intervals for at least 18 months after the tapering of the drugs”  Number of NCC patients: 227; Number of patients randomized: 206; Male: 137  Group A: 98; Group B: 108;  Patient’s characteristic: Children and adults  Lost to follow-up: 21  Seizure frequency: number of seizures (mean ± SD) in Group A: 2.3 ± 1.2 and in Group B: 2.5 ±1.6. |
|  | **Inclusion criteria:**  Fulfilling Del Brutto’s probable criteria, patients with seizures as initial symptom, no feature suggestive of a progressive neurological deficit, no evidence of persistent raised intracranial pressure or any active systemic disease, fulfilling criteria for SEL on CT, only patients with complete lesion resolution or calcified residue at repeat CT scan (3-6 months) were included;  **Exclusion criteria:**  Persistence of lesion at 3-6 months follow-up CT |
| Interventions | **Group A:**  AED: Carbamazepine or Phenytoin depending on affordability  Treatment duration: 6 months tapered over the next 6 weeks  **Group B:**  AED: Carbamazepine or Phenytoin depending on affordability  Treatment duration: 24 months tapered over the next 6 weeks |
| Outcomes | **Seizure recurrence**  Group A: 16 patients Group B: 13 patients **Calcifications** Group A: 33 patients Group B: 46 patients **Complete cyst resolution**  Group A: 65 patients Group B: 62 patients **Partial cyst resolution** Group A: 5 patients Group B: 6 patients |

**PICO 2**

| **Garg et al. 2006** **(4)** | |
| --- | --- |
| Study characteristics | India - Department of Neurology, King George Medical University, Uttar Pradesh  Randomized, double blind placebo-controlled trial  Duration: February 2004 – February 2005 |
| Participants | Diagnostic criteria: Rajshekhar et al. 1993  Follow up: “Patients were followed up at monthly interval at least for 9 months. Followed up CT scan were performed in all patients of both groups after 6 months.”  Number of patients: 60, Male: 39  Group A: 30; Group B:30;  Patient’s characteristic: Children and adults  Lost to follow-up: 0 |
|  | **Inclusion criteria:**  “Clinical and CT criteria given by Rajshekhar et al., for solitary cysticercus granuloma were used for inclusion of the patients. These criteria were new-onset seizure (14 days), no evidence of increased intracranial pressure, no evidence of neurological deficits, no evidence of other systemic illness; CT scan showing a single enhancing CT lesion of less than 20 mm in maximal dimensions.”  **Exclusion criteria:**  “presence of raised intracranial pressure, neurological deficits (other than Todd’s palsy), prior administration of anti-cysticercal treatment (albendazole) and history of peptic ulcer disease.” |
| Interventions | **Group A:**  Treatment: anti-epileptic monotherapy with  prednisolone  Prednisolone oral, 1 mg/kg/day for 10 days, followed by tapering over the next 4 days  **Group B:**  Treatment: anti-epileptic monotherapy along with placebo  All patients were administered antiepileptic drug monotherapy (phenytoin or carbamazepine) |
| Outcomes | **Seizure recurrence**  Group A: 4 patients Group B: 14 patients **Cyst resolution**  Group A: 16 patients Group B: 14 patients |

| **Kishore et al. 2007** **(5)** | |
| --- | --- |
| Study characteristics | India - Department of Neurology, Institute of Medical Sciences, Banaras Hindu University  Open label randomized prospective trial  Duration: NA |
| Participants | Diagnostic criteria: Rajshekhar et al. 1993  Follow up: “Follow up CT scan was performed immediately after  8-10 weeks of the initial CT scan.” “Patients were followed up for 1 year for seizure recurrence.”  Number of patients: 100, Male: 61  Group A: (50 patients, remaining 45); Group B: (50 patients, 47 remaining);  Patient’s characteristic: Children and adults  Lost to follow-up: 8 |
|  | **Inclusion criteria:**  **“**The inclusion criteria (i) Clinical Criteria:  Seizure (partial of generalized) should be the initial symptom, there should be no features of persistent raised intracranial pressure, there should be no history of progressive neurological deficit and there should be no evidence of an active systemic disease. (ii) The CT Criteria: CT scan should only show a solitary, contrast enhancing lesion, the lesion should measure less than 20 mm in maximal dimension and edema may or not be present, but is not severe enough to produce a shift of the midline structures as proposed by Rajshekher et al in favor of solitary neurocysticercosis.”  **Exclusion criteria:** NA |
| Interventions | **Group A:**  Treatment: antiepileptic drugs with oral prednisolone in a dose of 1 mg/kg body weight for 7 days and tapering off dose in next 3 days  **Group B:**  Treatment: AED + Placebo  Carbamazepine (10 mg/kg/day) in majority of the patients. Phenytoin (5-7mg/kg/day) was also given in few. |
| Outcomes | **Seizure recurrence**  Group A: 5 patients Group B: 12 patients **Calcifications** Group A: 2 patients Group B: 4 patients **Complete cyst resolution**  Group A: 32 patients Group B: 24 patients **Partial cyst resolution** Group A: 11 patients Group B: 8 patients |
| **Mall et al. 2003** **(6)** | |
| Study characteristics | India - Department of Neurology of Chhatrapati Shahuji Medical University, Lucknow  Open label randomized prospective follow-up trial  Duration: October 2001 – September 2002 |
| Participants | Diagnostic criteria: "fulfilled the diagnostic criterion of solitary cysticercus granuloma"  Follow up: “Fortnightly intervals for the first 2 months, and subsequently at monthly interval for 6 months. Follow-up CT scans were performed in all patients of both the groups after 1 and 6 months.”  Number of patients: 108 consecutive patients, 97 remained, Male: 56  Group A: 49; Group B:48;  Patient’s characteristic: Children and adults  Lost to follow-up: 6 (+5 excluded) |
|  | **Inclusion criteria:**  “The diagnostic criteria for inclusion were new-onset seizures; minimal or no neurologic deficit; no evidence of increased intracranial pressure; no evidence of other systemic disease, and a cranial CT scan showing an single enhancing CT lesion (diameter < 20mm). All included patients had a seizure disorder of ≤ 10 days‘ duration.”  **Exclusion criteria:**  “The exclusion criteria were appearance of increased intracranial pressure, persistent focal neurologic deficit, and/or enlargement of CT lesions in follow-up CT scan.” |
| Interventions | **Group A:**  Treatment: oral prednisolone along with antiepileptic monotherapy;  prednisolone oral (1 mg/kg/day (single-dose administration for 10 days), followed by tapering over the next 4 days, a 20% reduction each day  **Group B:**  Treatment: antiepileptic monotherapy;  Carbamazepine or sodium phenytoin |
| Outcomes | **Seizure recurrence**  Group A: 3 patients (six month) Group B: 13 patients (six month)  **1 months:**  **Complete cyst resolution**  Group A: 25 patients Group B: 11 patients  **Partial cyst resolution**  Group A: 14 patients  Group B: 9 patients  **6 months:**  **Calcifications**  Group A: 3 patients  Group B: 5 patients  **Complete cyst resolution**  Group A: 43 patients  Group B: 25 patients  **Partial cyst resolution**  Group A: 2 patients  Group B: 8 patients |

| **Singla et al. 2011** **(7)** | |
| --- | --- |
| Study characteristics | India - Department of Neurology, Postgraduate Institute of Medical Education & research, Chandigarh  Randomized, double-blind placebo controlled trial  Duration: July 2007 – December 2008 |
| Participants | Diagnostic criteria: "fulfilling previously validated criteria for SCG"  Follow up: 3 months (CT) and 6 months (MRI) for Cystic resolution and 9 months for Seizures  Number of patients: 148, Male: 104;  Group A: 73 after lost to follow up 62; Group B: 75 after lost to follow up 63;  Patient’s characteristic: Children and adults  Lost to follow-up: 23 |
|  | **Inclusion criteria:**  “new-onset seizures (<15 days duration) and imaging (either MRI or CT) fulfilling previously validated criteria for SCG”  **Exclusion criteria:**  “Patients were excluded if they demonstrated calcific lesions on imaging; evidence of central nervous system, pulmonary or systemic tuberculosis or concomitant systemic disease; positive human immunodeficiency virus serology; were pregnant women; had received prior anti-cysticercal treatment or corticosteroids; or were unwilling to enter the trial or follow the trial protocols or were unable to provide informed consent.” |
| Interventions | **Group A:**  Treatment: AED + Corticosteroids  Intervention comprised prednisolone (Ind-Swift, Town Baddi, Himachal Pradesh, India) (three tablets of 20 mg each for subjects >40 kg and two tablets of 20 mg each for those ≤40 kg) for 2 weeks followed by tapering doses over 4 days.  **Group B:**  Treatment: AED + Placebo;  Placebo tablets prepared were identical in shape, size, strength, color, and packing  All subjects received AEDs [either carbamazepine (12 tablets 15 mg/kg/day, dose increased to 30 mg/kg/day in case of recurrent seizures) or phenytoin (3–6 mg/kg/day)] |
| Outcomes | **Seizure recurrence**  Group A: 16 patients Group B: 19 patients **Calcifications** Group A: 8 patients Group B: 6 patients **Complete cyst resolution (3 months)**  Group A: 27 patients Group B: 23 patients **Complete cyst resolution (6 months)** Group A: 28 patients Group B: 21 patients |
