# Supplementary material for: The effectiveness of anti-inflammatory and anti-seizure medication for individuals with single enhancing lesion neurocysticercosis: A meta-analysis and expert group-based consensus recommendations
Source: PLoS Negl Trop Dis. 2021 Mar 31;15(3):e0009193. doi: 10.1371/journal.pntd.0009193 (PMC8057605; doi:10.1371/journal.pntd.0009193)
Supplement: S4 Table — Summary of included studies. (DOCX) [file pntd.0009193.s034.docx]

**S4 Table. Overview of main characteristics of included studies.** Summary of included studies.

**PICO 1**

| Author & year | Place | Study design | Intervention/ number of patients | Cortico-steroids | AEDs | Follow-up | Outcome – seizure recurrence | Outcome – cyst resolution |
| --- | --- | --- | --- | --- | --- | --- | --- | --- |
| *Gupta et al. 2002*  *(1)* | New Delhi, India | Randomized trial | Group A:  AEDs for 6 months  (41 patients)  Group B:  AEDs for 1 year (40 patients)  A total: 81 patients | - | Not available | 1 year after stopping AEDs (1,5 or 2 years)  repeat CT/MRI: 3months and/ or 6 months | Group A:  5 patients (after 4.3+1.52 months of stopping treatment)  Group B:  5 patients (after 2.2+1.48 months of stopping treatment) | Calcifications  Group A: 4 patients  Group B: 4 patients  (at 6 months follow up) |
| *Thussu et al. 2002*  *(2)* | Chandigarh, India | Randomized trial | Group A:  AEDs for 6 months (47 patients)  Group B:  AEDs for 2 years (26 patients)  A total: 73^[[1]](#footnote-1)^ | - | Carbamazepine (n=38),  Phenytoin (n=35) | 1 year after stopping AED (1,5 or 3 years)  repeat CT: at the end of 3 months | Group A: 8 patients  Group B: 3 patients | Complete cyst resolution Group A: 25 patients  Group B: 14 patients  Calcifications  Group A: 22 patients  Group B: 12 patients  (CT scans at the end of 3 months) |
| *Verma et al. 2006*  *(3)* | Varanasi,  India | Randomized trial | Group A:  AEDs for 6 months (98 patients)  Group B:  AEDs for 2 years (108 patients)  A total: 227 patients and 21 patients lost to follow-up | - | Carbamazepine (n=176),  Phenytoin (n=51) | at least 18 months after AED tapering (2 or 3,5 years)  repeat CT: at the interval of 3–6 months | Group A: 16 patients  Group B: 13 patients | Complete cyst resolution Group A: 65 patients  Group B: 62 patients  Partial cyst resolution  Group A: 5 patients  Group B: 6 patients  Calcifications  Group A: 33 patients  Group B: 46 patients  (during the follow-up period of 18 months) |

**PICO 2**

| Author & year | Place | Study design | Intervention/ number of patients | Corticosteroids | AEDs | Follow-up | Outcome – seizure recurrence | Outcome – cyst resolution |
| --- | --- | --- | --- | --- | --- | --- | --- | --- |
| *Garg et al. 2006*  *(4)* | Lucknow, India | Randomized double blind placebo- controlled trial | Group A:  AED + corticosteroids  (30 patients)  Group B:  AED + placebo  (30 patients) | prednisolone (oral)  1 mg/kg body weight/day  10 days + tapering off 4 days | phenytoin or carbamazepine  monotherapy | 6 months, 9 months | Group A:  4 patients (1 normal CT, 3 abnormal CT scans)  Group B:  14 patients (3 normal CT,11 abnormal CT scans)  (9 months) | Complete cyst resolution:  Group A: 16 patients  Group B: 14 patients  (6 months) |
| *Kishore et al. 2007*  *(5)* | Varanasi,  India | Open label randomized prospective trial | Group A:  AED + corticosteroids  (50 patients, remaining 45)  Group B:  AED + placebo  (50 patients, 47 remaining) | prednisolone (oral)  1 mg/kg body weight/day  7 days + tapering off 3 days | carbamazepine (10 mg/kg/day)  phenytoin (5-7 mg/kg/day) | 8-12 weeks,  12 months | Group A:  5 patients (including 2 patients, where AED dose was built up)  Group B:  12 patients (including 7, where dose was built up)  (12 months) | Complete cyst resolution  Group A: 32 patients  Group B: 24 patients  Partial cyst resolution  Group A: 11 patients  Group B: 8 patients  Calcifications  Group A: 2 patients  Group B: 4 patients  (8-12 weeks) |
| *Mall et al. 2003*  *(6)* | Lucknow, India | Open label randomized prospective follow-up trial | Group A:  AED + corticosteroids  (49 patients)  Group B:  AED  (48 patients) | prednisolone (oral)  1 mg/kg/day single-dose administration for 10 days followed by tapering over the next 4 days, a 20% reduction each day | carbamazepine or sodium phenytoin | 1 month,  6 months | Group A:  3 patients  Group B:  13 patients  (6 months) | Complete cyst resolution  Group A: 25 patients  Group B: 11 patients  Partial cyst resolution  Group A: 14 patients  Group B: 9 patients  (1 month)  Complete cyst resolution  Group A: 43 patients  Group B: 25 patients  Partial cyst resolution  Group A: 2 patients Group B: 8 patients  Calcifications  Group A: 3 patients  Group B: 5 patients  (6 months) |
| *Singla et al. 2011*  *(7)* | Chandigarh, India | Randomized, double-blind, placebo-controlled trial | Group A:  AED + corticosteroids  (73 patients, remaining 62 patients, at 6 month MRI: 60 patients)  Group B:  AED + placebo  (75 patients, remaining 63, at 6 month MRI: 54 patients) | prednisolone  (oral)  40-60 mg/ day^[[2]](#footnote-2)^) for 2 weeks followed by tapering doses over 4 days | carbamazepine (12 tablets 15mg/kg/day, dose increased to 30 mg/kg/ day in case of recurrent seizures) or phenytoin (3-66 mg/kg/day) | 3 months, 6 months,  9 months | Group A:  16 patients  Group B:  19 patients  (9 months) | Complete cyst resolution  Group A: 27 patients  Group B: 23 patients  Calcifications  Group A: 8 patients  Group B: 6 patients  (3 months)  Complete cyst resolution  Group A: 28 patients  Group B: 21 patients  (6 months) |

1. „A repeat plain and contrast enhanced CT scan was done on follow up at 12 weeks.(…) Cases who had persistence of the lesion, were put on albendazole and excluded from the study.” [↑](#footnote-ref-1)
2. three tablets of 20 mg each for subjects >40 kg and two tablets of 20 mg each for those <40 kg [↑](#footnote-ref-2)
