# Supplementary material for: The effectiveness of anti-inflammatory and anti-seizure medication for individuals with single enhancing lesion neurocysticercosis: A meta-analysis and expert group-based consensus recommendations
Source: PLoS Negl Trop Dis. 2021 Mar 31;15(3):e0009193. doi: 10.1371/journal.pntd.0009193 (PMC8057605; doi:10.1371/journal.pntd.0009193)
Supplement: S5 Table — Descriptions of the judgments made for the included studies. (DOCX) [file pntd.0009193.s035.docx]

**S5 Table. Risk of bias assessment.** Descriptions of the judgments made for the included studies.

**Risk of bias – anti-epileptic treatment**

| Generation of allocation sequence | Three out of four studies describe a random component used in the sequence generation (e.g. random number table, coin tossing) (2, 3, 8). Gupta et al. does not further elaborate on their method of randomization (1). |
| --- | --- |
| Allocation concealment | In none of the studies any method of allocation concealment is mentioned, thus selection bias remains unclear. |
| Blinding | We judged performance bias as high in all of the included studies as knowledge of the allocated intervention could have been foreseen by both personnel and participants due to the different length of treatment and as no placebo was used in any studies for blinding purposes.  Blinding of outcome assessment was not reported on by any study, thus leading to an unclear detection bias. |
| Addressing incomplete outcome data | Two studies were found to have no missing outcome data and were therefore judged as low risk of bias (1, 2). Two studies, however, were classified as unclear risk of bias: in the study of Singhi and Verma insufficient information was provided to permit a judgment of low or high risk of attrition bias. Singhi reports on nine patients and Verma on 21 patients lost-to follow up, but it does not become clear how this was adjusted for, although Verma is mentioning an intention-to-treat analysis (3, 8). |
| Other biases | => See main manuscript |

**Risk of bias – anti-inflammatory treatment**

| Generation of allocation sequence | Three out of the four studies clearly stated the method used for randomization (4, 6, 7), whereas Kishore did not state it other than in the title of the article (5). The methods used included referring to a random number table (4, 6), and using a computer random number generator (7). For the articles where the method for randomization was stated we find it to be a low risk for selection bias (4, 6, 7), whereas in the study of Kishore et al. the risk for selection bias is unclear due to the method used for randomization not being stated (5). |
| --- | --- |
| Allocation concealment | Singla is the only one of the four articles that states a method for allocation concealment. As the randomization where “administered by one study team member (BM), who had no other role in the conduct of the trial.” we find the risk for allocation concealment bias to be low (7). In all of the other three articles, there is an unclear risk for allocation concealment bias as the methods used for concealment have not been stated (4-6). |
| Blinding | We only find one of the articles to have a low risk for performance and detection bias. Although methods of blinding participants and study personnel is not being stated we find that Singla have a low risk for performance and detection bias as “study team members and patients were blinded to the randomization code and group-labeling. The code was opened upon completion of follow-up period of the trial.” (7).  None of the other articles mentions blinding of participants or study personnel apart from Kishore reporting that “visual analysis was performed by an independent observer (radiologist not familiar with the clinical course of the patient)” (5).  We also find Mall to have a high risk for performance bias due to no placebo being used (6). |
| Addressing incomplete outcome data | Two studies reported on patients excluded due to loss of follow-up (5, 6). As no statistical method to adjust to this was mentioned we considered them as high risk of attrition bias. Singla et al. on the contrary do report applying an intention-to-treat analysis to account for the patients lost to follow-up. Nevertheless, concerning the outcome of cyst resolution, a discrepancy between the number of patients reported as lost (23) and the number of patients receiving a CT at 3 months (116) and MRI at 6 months (114) out of a total of 148 patients remains unclear (7). Therefore, we judge the risk for attrition bias to be unclear. |
| Selective reporting | None of the articles included had protocols available for comparison with the published conduct of the studies we therefore judge the risk of reporting bias to be unclear for all four of the studies. |
| Other biases | We find that the limited information on study design and methods provided by the authors of the studies restricts the possibility to asses any further substantial risk of bias. |

**References**

1. Gupta M, Agarwal P, Khwaja GA, Chowdhury D, Sharma B, Bansal J, et al. Randomized prospective study of outcome of short term antiepileptic treatment in small single enhancing CT lesion in brain. Neurol India. 2002;50(2):145-7.

2. Thussu A, Arora A, Prabhakar S, Lal V, Sawhney IM. Acute symptomatic seizures due to single CT lesions: how long to treat with antiepileptic drugs? Neurol India. 2002;50(2):141-4.

3. Verma A, Misra S. Outcome of short-term antiepileptic treatment in patients with solitary cerebral cysticercus granuloma. Acta neurologica Scandinavica. 2006;113(3):174-7.

4. Garg RK, Potluri N, Kar AM, Singh MK, Shukla R, Agrawal A, et al. Short course of prednisolone in patients with solitary cysticercus granuloma: a double blind placebo controlled study. J Infect. 2006;53(1):65-9.

5. Kishore D, Misra S. Short course of oral prednisolone on disappearance of lesion and seizure recurrence in patients of solitary cysticercal granuloma with single small enhancing CT lesion: an open label randomized prospective study. J Assoc Physicians India. 2007;55:419-24.

6. Mall RK, Agarwal A, Garg RK, Kar AM, Shukla R. Short course of prednisolone in Indian patients with solitary cysticercus granuloma and new-onset seizures. Epilepsia. 2003;44(11):1397-401.

7. Singla M, Prabhakar S, Modi M, Medhi B, Khandelwal N, Lal V. Short-course of prednisolone in solitary cysticercus granuloma: a randomized, double-blind, placebo-controlled trial. Epilepsia. 2011;52(10):1914-7.

8. Singhi PD, Dinakaran J, Khandelwal N, Singhi SC. One vs. two years of anti-epileptic therapy in children with single small enhancing CT lesions. J Trop Pediatr. 2003;49(5):274-8.
